# GADL1 is a multifunctional decarboxylase with tissue-specific roles in β-alanine and carnosine production

**DOI:** 10.1126/sciadv.abb3713

**Published:** 2020-07-17

**Authors:** Elaheh Mahootchi, Selina Cannon Homaei, Rune Kleppe, Ingeborg Winge, Tor-Arne Hegvik, Roberto Megias-Perez, Christian Totland, Floriana Mogavero, Anne Baumann, Jeffrey Colm Glennon, Hrvoje Miletic, Petri Kursula, Jan Haavik

**Affiliations:** 1 Department of Biomedicine, University of Bergen, Bergen, Norway.; 2 Division of Psychiatry, Haukeland University Hospital, Bergen, Norway.; 3 Department of Chemistry, University of Bergen, Bergen, Norway.; 4 Norwegian Geotechnical Institute, Oslo, Norway.; 5 Department of Cognitive Neuroscience, Donders Institute for Brain Cognition and Behavior, Radboud University Medical Center, Nijmegen, Netherlands.; 6 Conway Institute of Biomolecular and Biomedical Research, School of Medicine, University College Dublin, Belfield, Dublin 4, Ireland.; 7 Department of Pathology, Haukeland University Hospital, Bergen, Norway.; 8 Faculty of Biochemistry and Molecular Medicine, University of Oulu, Oulu, Finland.

## Abstract

Carnosine and related β-alanine–containing peptides are believed to be important antioxidants, pH buffers, and neuromodulators. However, their biosynthetic routes and therapeutic potential are still being debated. This study describes the first animal model lacking the enzyme glutamic acid decarboxylase–like 1 (GADL1). We show that Gadl1^−/−^ mice are deficient in β-alanine, carnosine, and anserine, particularly in the olfactory bulb, cerebral cortex, and skeletal muscle. Gadl1^−/−^ mice also exhibited decreased anxiety, increased levels of oxidative stress markers, alterations in energy and lipid metabolism, and age-related changes. Examination of the GADL1 active site indicated that the enzyme may have multiple physiological substrates, including aspartate and cysteine sulfinic acid. Human genetic studies show strong associations of the *GADL1* locus with plasma levels of carnosine, subjective well-being, and muscle strength. Together, this shows the multifaceted and organ-specific roles of carnosine peptides and establishes Gadl1 knockout mice as a versatile model to explore carnosine biology and its therapeutic potential.

## Teaser

Mice lacking the enzyme GADL1 have reduced levels of carnosine and anserine peptides and increased oxidative stress markers.

## INTRODUCTION

Carnosine (β-alanyl-l-histidine) is one of several dipeptides of β-alanine and histidine that are found in high concentrations in vertebrate tissues, particularly in the brain and skeletal muscle (SKM) tissues. In humans, carnosine and acetylcarnosine are most abundant. In contrast, many animals mainly synthesize the related β-alanine–containing peptides anserine or ophidine/balenine through methylation of the imidazole moiety of carnosine in the 1 or 3 position (*1*, *2*). Here, we refer to this family of related peptides as “carnosine peptides.”

Carnosine peptides may have multiple biological functions, including calcium regulation, pH buffering, metal chelation, and antioxidant effects (*1*). β-Alanine, as well as its dipeptide derivatives, may also be neurotransmitters or neuromodulators in the central nervous system, especially in olfactory bulb (OB). In mammals, including mice, only two tissues have carnosine concentrations in the millimolar range, i.e., SKM and OB (*1*, *3*). Various disorders and dysfunctions have been linked to alterations in β-alanine and carnosine metabolism. In β-alaninemia and carnosinemia, decreased degradation of these compounds is associated with neurological symptoms (*4*, *5*). Conversely, it has been suggested that some age-related and neurological diseases, e.g., Alzheimer’s disease, Parkinson’s disease, multiple sclerosis (MS), and cancer, as well as diabetes complications, may benefit from carnosine supplementation (*6*).

β-Alanine supplementation increases carnosine content and improves contractility and muscle performance of human and rodent SKM, especially during short exercise intervals (*7*, *8*). These findings have led to the widespread use of dietary β-alanine supplements, particularly among athletes and soldiers (*9*). β-Alanine and carnosine dietary supplementation have also shown some promising effects in the treatment of depression, anxiety, and autism symptoms in humans (*10*) and animal models (*11*, *12*).

As most carnosine peptides are hydrolyzed after oral ingestion, either in enterocytes or in the blood circulation, the body mainly depends on de novo synthesis of these peptides. Dietary supplementation has side effects but requires a daily intake of several grams to increase muscle β-alanine and carnosine content and physical performance (*13*). The lack of suitable model systems and the need to supply pharmacological quantities of carnosine peptides have caused some uncertainty regarding the mechanisms of action and safety of these compounds. A more stable, synthetic analog of carnosine, carnosinol, was recently shown to protect against metabolic dysregulation and oxidative damage (*14*).

Synthesis of β-alanine appears to be a rate-limiting factor for carnosine peptide levels in mammals (*15*). In a large genome-wide association (GWA) study on plasma metabolites, it was estimated that 86% of the variation in carnosine levels could be attributed to genetic factors, making this peptide the most heritable metabolite among all compounds examined (*16*). Still, uncertainty remains regarding the identity of the genes and proteins involved in its synthesis. Carnosine synthase 1 (encoded by *CARNS1*) produces carnosine from β-alanine and histidine and homocarnosine from γ-aminobutyric acid (GABA) and histidine. In animal tissues, β-alanine may be produced by reductive degradation of uracil (*17*). In contrast, some bacteria produce β-alanine by α-decarboxylation of aspartic acid (Asp), catalyzed by aspartate decarboxylase with the aid of a covalently bound pyruvoyl cofactor (*18*). In insects, an analogous vitamin B6 [pyridoxal phosphate (PLP)]–dependent enzyme l-aspartate-α-decarboxylase has evolved by convergent evolution (*19*). The formation of carnosine in the mouse SKM is dependent on vitamin B6, implicating a PLP-dependent enzyme in its synthesis in animals as well (*20*).

On the basis of its sequence similarity to glutamic acid decarboxylase (GAD), the PLP-dependent enzyme GAD-like protein 1 (GADL1; acidic amino acid decarboxylase) has been assigned a possible role in GABA synthesis and to be associated with lithium response in bipolar patients (*21*). Although this genetic association was not replicated in other clinical samples (*22*), these findings have triggered interest in the in vivo investigation of GADL1 function and biochemical properties. As cysteine sulfinic acid (CSA) is a substrate of GADL1, it was suggested that GADL1 is involved in the biosynthesis of hypotaurine and taurine (*23*). However, it has also been reported that purified human (*23*) and mouse (*24*) GADL1 can synthesize β-alanine from Asp, although at very low rates in vitro. This is consistent with recent crystallographic studies, showing that GADL1 shares structural features with both CSA decarboxylase (CSAD) and Asp decarboxylase (*25*). Moreover, a human genetic association study demonstrated that single-nucleotide polymorphisms (SNPs) in the *GADL1* intron are strongly associated with blood levels of acetylcarnosine (*26*) that, in turn, are strongly correlated with carnosine levels (*2*). On the basis of these observations, we hypothesized that GADL1 could be involved in β-alanine and carnosine production in mammalian tissues.

Here, we describe the first *Gadl1* knockout (KO) mouse model and demonstrate an organ-specific role of GADL1 in the biosynthesis of carnosine peptides and protection against oxidative stress, particularly in the OB and SKM. To understand the substrate specificity of GADL1, we compared the three-dimensional (3D) structures of mouse GADL1 and related enzymes. Last, we investigate the association between common genetic variants in enzymes and transporters involved in carnosine homeostasis with multiple human traits and diseases and present an initial behavioral characterization of the *Gadl1* KO mouse.

## RESULTS

### Mice lacking GADL1 show age-related changes

**Fig. 1:**
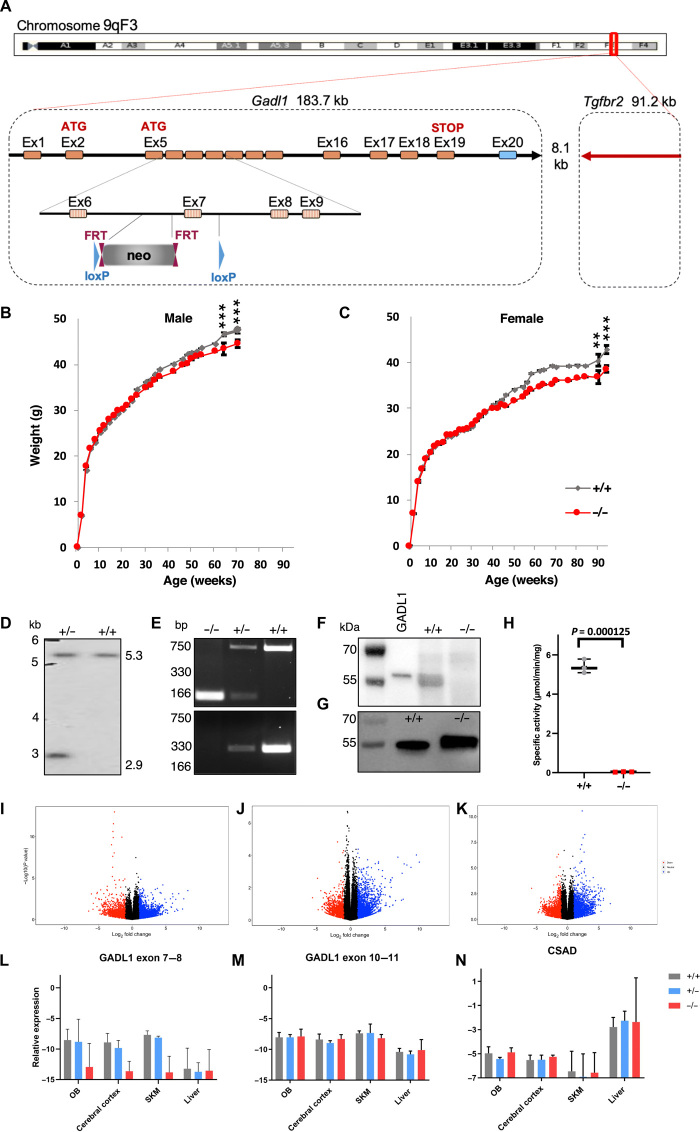
Generation and characterization of GADL1 KO mice. (**A**) Targeting strategy for knocking out exon (Ex) 7 of the mouse *Gadl1* locus on chromosome 9. *Gadl1* coding sequences (hatched rectangles), noncoding exon portions (blue rectangle), and chromosome sequences (orange rectangles) are represented. The neomycin (neo)–positive selection cassette is indicated between loxP sites (blue triangles) and Flippase recognition target (FRT) sites (plum triangles). (**B** and **C**) Growth curves of *Gadl1*^+/+^ (*n* = 4 to 34) and *Gadl1*^−/−^ mice (*n* = 4 to 40), presented as means ± SD. Differences between genotypes were significant; *P* = 0.0008 (64 weeks) and *P* = 0.0005 (70 weeks) for males and *P* = 0.0084 (90 weeks) and *P* = 0.0001 (94 weeks) for females, respectively. (**D**) Southern blot analysis of genomic DNA from *Gadl1*^+/+^ and *Gadl1*^−/−^. (**E**) Genotyping of the offspring from intercrosses of *Gadl1*^+/−^ mice by polymerase chain reaction (PCR). The DNA band at 166 base pair (bp) is the KO allele (primer 3), while bands at 330 bp (primer 2 and 3) and 750 bp (primer 1) are the wild-type alleles. (**F**) Representative Western blots of OB samples from *Gadl1*^+/+^ and *Gadl1*^−/−^ mice (34 weeks old, female) using anti-GADL1 antibody. Positive control was recombinant His-tagged GADL1 (2 ng; lane 1). (**G**) Western blot of recombinant His-tagged truncated Gadl1^+/+^ and Gadl1^−/−^. (**H**) Enzyme activity toward CSA of recombinant His-tagged truncated *Gadl1*^+/+^ and *Gadl1*^−/−^; *P* < 0.001. (**I** to **K**). RNA expression levels (volcano plots) in OB tissue. (I) *Gadl1*^+/+^-to-*Gadl1*^+/−^ ratio, (J) *Gadl1*^+/+^-to-*Gadl1*^−/−^ ratio, and (K) *Gadl1*^−/−^-to-*Gadl1*^+/−^ratio. (**L** to **N**) Quantitative reverse transcription PCR (qRT-PCR) analysis of normalized mRNA expression in OB, brain, SKM, and liver tissues from 35-week-old female *Gadl1*^+/+^ (gray)*, Gadl1*^+/−^ (blue), and *Gadl1*^−/−^ (red) mice for (L) *Gadl1* exons 7 and 8, (M) *Gadl1* exons 10 and 11, and (N) CSAD. *n* = 3 for each genotype. Presented on an Ln *y* scale as mean of 2^Δ*C*t^ and upper limit (95%).

*Gadl1*^−/−^ (null) mice were generated using *Cre*/loxP technology. Because of the proximity of the *Gadl1* gene to the *Tgfbr2* gene that encodes a receptor with possible effects on growth and survival, we applied a conservative knocking out strategy, where only *Gadl1* exon 7, coding for part of the PLP-binding active site of GADL1, was deleted (Fig. 1A). *Gadl1*^+/+^, *Gadl1*^+/−^, and *Gadl1*^−/−^ mice were successfully generated in the expected Mendelian ratios by breeding from *Gadl1*^+/−^ mice. We observed no obvious physical abnormalities. Overall activity and feeding behavior, as well as initial growth curves, were similar across all genotypes. However, after 30 weeks of age, compared to *Gadl1*^+/+^ mice, female and male *Gadl1*^−/−^ mice exhibited relative growth retardation, compatible with age-related and possible degenerative changes (Fig. 1, B and C).

Genomic DNA sequencing and Southern blot analyses confirmed the elimination of exon 7 (Fig. 1, D and E). However, quantification of *Gadl1* mRNA from SKM and OB revealed that some *Gadl1* mRNA species could be detected across all genotypes. RNA sequencing showed that *Gadl1*^−/−^ mice lacked *Gadl1* exons 7 and 8. This was confirmed using quantitative reverse transcription polymerase chain reaction (qRT-PCR) of individual exons (see below). Bioinformatic analyses showed that the deletion of exons 7 and 8 was coupled to the generation of a new RNA splicing site in the mutated mice (fig. S1).

Western blotting confirmed that GADL1 protein was present in *Gadl1*^+/+^ and *Gadl1*^+/−^ but not in *Gadl1*^−/−^ mice. In OB extracted from *Gadl1*^+/+^ mice, GADL1 appeared as a wide band, with an estimated molecular mass of 55 to 59 kDa (Fig. 1F). This corresponds to several predicted protein variants with 502 to 550 amino acids (*24*). Since exons 7 and 8 encode amino acids involved in cofactor binding, GADL1 lacking these amino acids was predicted to be enzymatically inactive. This was confirmed by expressing the protein lacking exons 7 and 8 in *Escherichia coli* and comparing it to the full-length protein (Fig. 1G and fig. S2). The mutant protein yield was only 17% compared to that of the full-length protein. Furthermore, the mutant protein was completely devoid of enzyme activity (Fig. 1H). This demonstrated that the elimination of gene function was successful and that the *Gadl1*^−/−^ mice did not have any residual GADL1 enzyme activity.

### Elimination of *Gadl1* alters the OB transcriptome

To determine the effects of GADL1 depletion on gene expression, we sequenced and compared OB mRNA levels from *Gadl1*^+/+^, *Gadl1*^+/−^, and *Gadl1*^−/−^ mice (Fig. 1, I to K). The top 25 up- and down-regulated genes (log_2_ fold change either less than −1 or greater than +1 and *P* ≤ 0.05) are shown in table S1 and fig. S3. Pathway enrichment analysis showed that the strongest affected group of genes is involved in drug metabolism [Kyoto Encyclopedia of Genes and Genomes (KEGG pathway mmu00983; *P* = 0.00016, *q* = 0.014] (Table 1) (*27*). This pathway includes the *Upb1* gene (β-ureidopropionase 1), a transcript with a twofold increase in *Gadl1*^−/−^ mice (*P* = 0.0154). UPB1 catalyzes the last step in the formation of β-alanine from pyrimidines. This is consistent with a compensatory mechanism for maintaining β-alanine synthesis in the absence of GADL1. Moreover, in OB of *Gadl1*^−/−^ mice, we observed increased transcript levels of multiple isoforms of carboxylesterase 1, cytochrome P450, and myeloperoxidase.

**Table 1:** Pathway enrichment analysis comparing *Gadl1*^−/−^ to *Gadl1*^+/+^. Green, up-regulated; yellow, down-regulated.

					**Drug** **metabolism**	**Retinol** **metabolism**	**Xenobiotics** **metabolism**	**Tryptophan** **metabolism**
				**KEGG ID**	mmu00983	mmu00830	mmu00980	mmu00380
**Gene ID**	**Abbr.**	**Name**	**Log_2_ fold**	***P* value**	0.00016	0.00022	0.00025	0.00045
14869	Gstp2	Glutathione *S*- transferase, pi 2	−4.2819	0.0374				
13897	Ces1e	Carboxylesterase 1E	4.9087	0.0040				
104158	Ces1d	Carboxylesterase 1D	3.8723	0.0131				
234564	Ces1f	Carboxylesterase 1F	2.6056	0.0193				
17523	Mpo	Myeloperoxidase	2.4247	0.0001				
394432	Ugt1a7c	uridine 5′-diphospho glucuronosyltransferase 1A7C	1.0524	0.0019				
103149	Upb1	β-Ureidopropionase	1.0121	0.0154				
27400	Hsd17b6	Hydroxysteroid (17-β) dehydrogenase 6	4.0829	0.0103				
13087	Cyp2a5	Cytochrome P450 2A5	3.9985	0.0129				
213043	Aox2	Aldehyde oxidase 2	3.4965	0.0260				
13086	Cyp2a4	Cytochrome P450 2A4	3.1973	0.0157				
13076	Cyp1a1	Cytochrome P450 1A1	3.0800	0.0010				
11522	Adh1	Alcohol dehydrogenase 1, class 1	1.7208	0.0144				
13107	Cyp2f2	Cytochrome P450 2F2	2.9664	0.0024				
12409	Cbr2	Carbonyl reductase 2	1.2641	0.0012				
11298	Aanat	Arylalkylamine *N*-acetyltransferase	−1.3632	0.0008				
15930	Ido1	Indoleamine 2,3-dioxygenase 1	2.4039	0.0192				
21743	Inmt	Indolethylamine *N*-methyltransferase	1.2043	0.0401				

**Table 2:** Male and female mice used in the metabolomic study.

**Genotype**	**Sex**	**Number (*n*)**	**Age** **(weeks ± SD)**
*Gadl1*^+/+^	Female	11	14.64 ± 7.85
*Gadl1*^−/−^	Female	11	14.27 ± 7.85
*Gadl1*^+/+^	Male	9	8.88 ± 3.98
*Gadl1*^−/−^	Male	10	8.40 ± 3.81

A gene ontology (GO) pathway analysis (*27*, *28*) of up- or down-regulated genes (log_2_ fold change either less than −1 or greater than +1 and *P* ≤ 0.05) showed statistical significant alterations in 24 different biological processes, where the most significant alteration involves drug catabolism (GO:0042737; *P* = 2.43 × 10^−7^, *q* = 0.0007). Furthermore, biological processes involving circadian rhythms and sleep cycles seemed to be altered. Dopamine receptors (*Drd1 to Drd3*) and adenosine A2a receptor (*Adora2a*) were the most consistently recurring genes throughout the GO analysis (table S2). It has been reported that the release of glutamate from terminals of carnosine containing olfactory neurons in OB glomeruli is modified by dopamine receptors (*29*). It is possible that the elimination of GADL1 and carnosine depletion also affected the expression of these modulatory transmitter receptors in the OB. Wu *et al.* (*30*) recently reported that GADL1 overexpression inhibited Potassium channel tetramerization domain containing 12 (KCTD12) expression. However, a comparison of *Gadl1*^−/−^ and *Gadl1*^+/+^ mice showed that RNA levels of KCTD12 were unaffected by the deletion of *Gadl1* (*P* = 0.62).

### Deletion of *Gadl1* perturbs carnosine metabolism

**Fig. 2:**
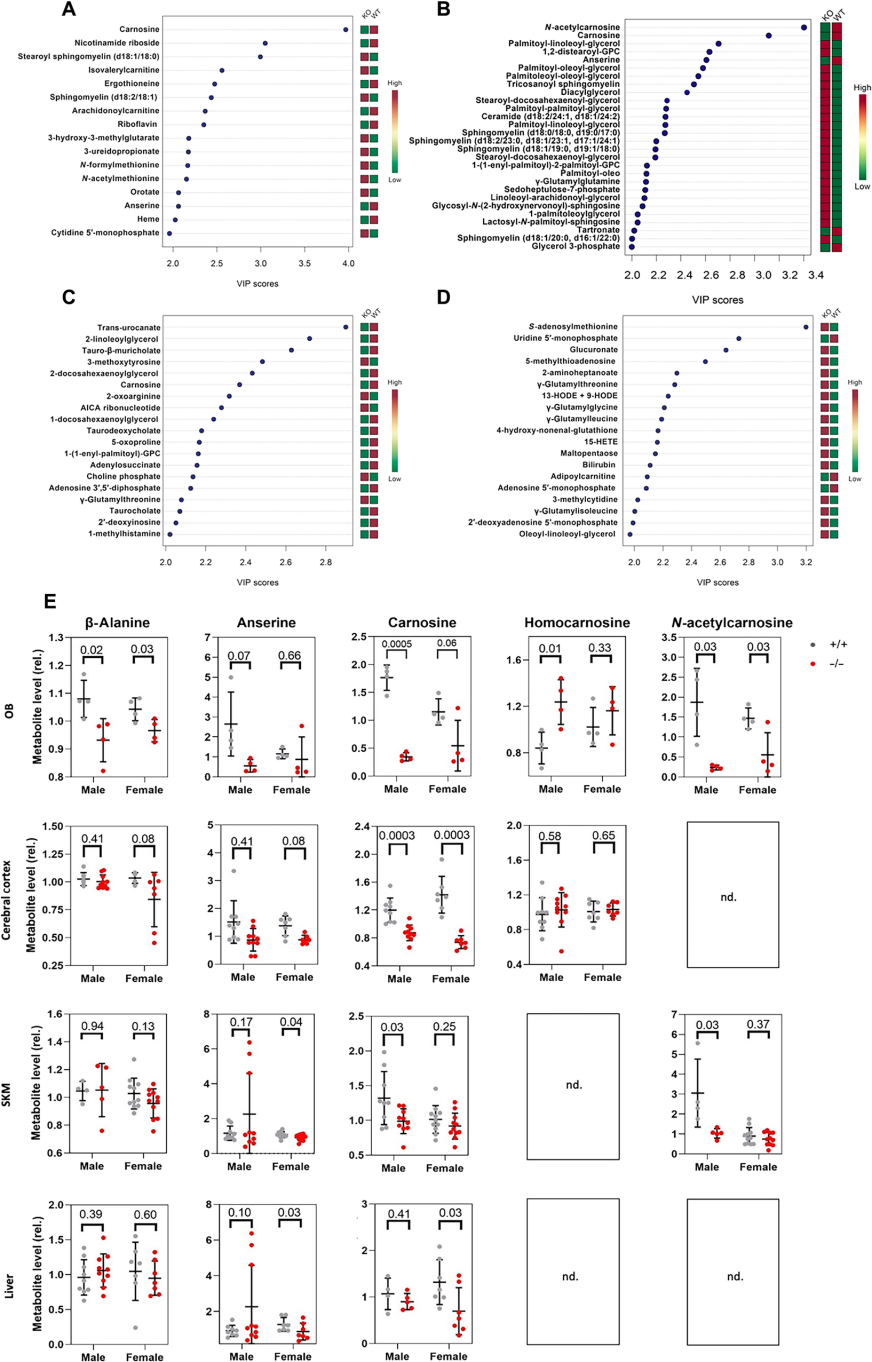
Tissue-specific effects on metabolite levels in *Gadl1*^−/−^mice. (**A** to **D**) Top significant features of metabolites based on VIP scores of >2 of component 1 of PLS-DA. Untargeted metabolic profiling of (A) cerebral cortex, (B) OB, (C) SKM, and (D) liver tissue samples from *Gadl1*^+/+^ (*n* = 20) and *Gadl1*^−/−^ (*n* = 21) mice. WT, wild-type. (**E**) The relative levels of β-alanine and carnosine derivatives in *Gadl1*^+/+^ (gray) and *Gadl1*^−/−^ (red) mouse tissue. nd., not determined.

To explore the biological function(s) of GADL1, we performed untargeted liquid chromatography–mass spectrometry (LC-MS) metabolomic analyses of eight different tissues from 20 *Gadl1*^+/+^ mice and 21 *Gadl1*^−/−^ mice that were matched across genotype and sex (Fig. 2). The number of identifiable metabolites varied between 541 and 720 in the cerebral cortex, OB, SKM, liver, cerebellum, heart, serum, and kidney. Partial least squares–discriminant analyses (PLS-DA) were used to evaluate metabolic differences between *Gadl1*^+/+^ and *Gadl1*^−/−^ mice. This allowed identification of suitable markers responsible for the metabolic differences by variable important projection (VIP) scores.

The metabolic features with VIP scores higher than 2, responsible for the separation in each tissue, are depicted in Fig. 2 (A to D). Carnosine peptides showed the strongest difference between the genotypes in the OB and the cerebral cortex (Fig. 2E). Carnosine was also among the most strongly affected metabolites in SKM but not in the liver (Fig. 2E). Although GADL1 has been postulated to synthesize GABA from glutamate, the levels of GABA and glutamate in *Gadl1*^+/+^ and *Gadl1*^−/−^ mice were similar in all tissues examined (fold difference of 0.88 to 1.15 for glutamate and 0.96 to 1.53 for GABA; *P* > 0.05). *Gadl1*^−/−^ mice had significantly reduced levels of β-alanine in OB but nonsignificant reduction in the cerebral cortex and SKM and unaltered levels in the liver. In contrast, carnosine, acetylcarnosine, and anserine were significantly depleted in brain and SKM tissues. A nonsignificant reduction (~10%) of β-alanine and carnosine peptides was observed in the serum and whole blood (fig. S4A), as well as in the kidney, cerebellum, and heart. Together, this is consistent with the observed tissue distribution of GADL1 and demonstrates its key role in the metabolic pathway of β-alanine and carnosine peptides.

### High-resolution magic angle spinning nuclear magnetic resonance spectroscopy confirms the depletion of carnosine in *Gadl1*^−/−^ mice

**Fig. 3:**
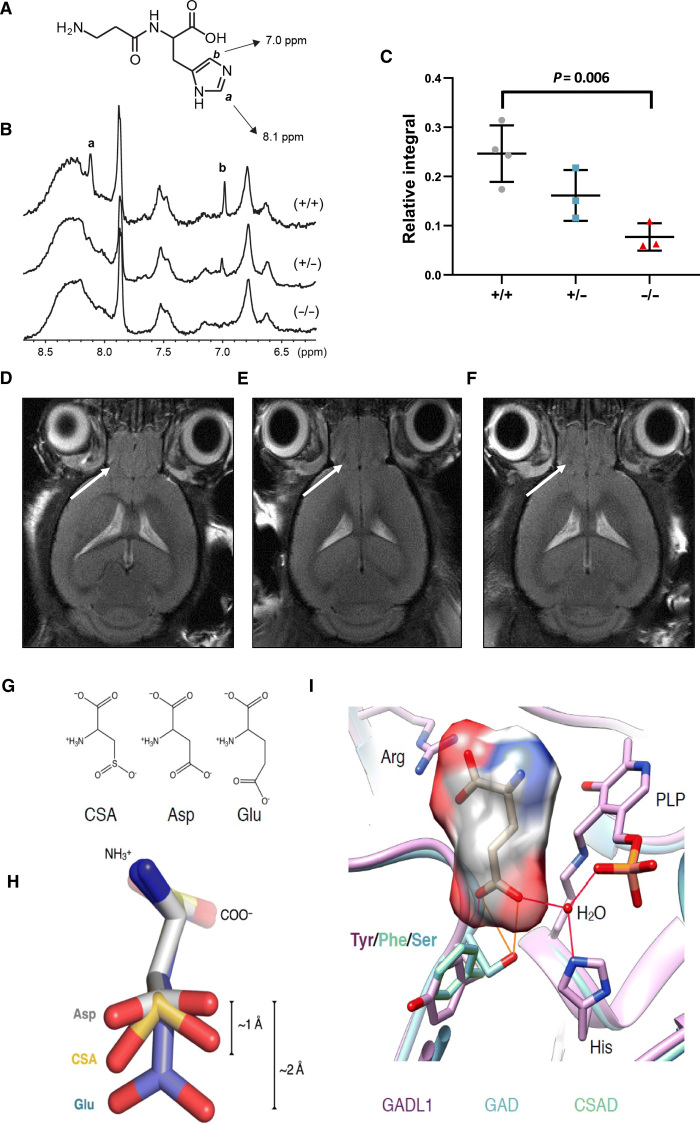
^1^H-NMR and MRI of mouse tissues and substrate specificity of GADL1. (**A** to **C**) Measurement of carnosine in intact OB tissue. (A) Chemical structure of carnosine. ppm, parts per million. (B) MAS ^1^H-NMR spectra of OB tissue samples from *Gadl1*^+/+^, *Gadl1*^+/−^, and *Gadl1*^−/−^ male mice (12 weeks). The two hydrogens of the imidazole ring in carnosine are marked a and b. (C) Relative integral based on NMR results, presented as means ± SD. (**D** to **F**) MRI of the brain in (D) *Gadl1*^+/+^, (E) *Gadl1*^+/−^, and (F) *Gadl1*^−/−^ mice. The arrow indicates the OB. (**G**) Chemical structure comparison of GADL1 substrates CSA, Asp, and Glu. (**H**) 3D substrate structures to show the size and shape differences of Glu compared to CSA and Asp. (**I**) Active sites of GADL1, GAD, and CSAD and the predicted mode of binding of Glu to GAD. The prediction is based on the complex between GAD and the inhibitor chelidonic acid (*63*).

To determine the role of GADL1 on carnosine content in intact tissue, we used fresh OB tissue where we found the highest expression GADL1. We performed high-resolution magic angle spinning (MAS) ^1^H–nuclear magnetic resonance (NMR) spectroscopy on OB from *Gadl1*^+/+^ (*n* = 4), *Gadl1*^+/−^ (*n* = 3), and *Gadl1*^−/−^ (*n* = 3) mice. Characteristic features of carnosine (Fig. 3A) could be identified in the aromatic regions of the spectrum, in accordance with the quantification in tissue extracts (Fig. 3B). Compared to *Gadl1*^+/+^, *Gadl1*^+/−^, and *Gadl1*^−/−^ mice had 34 and 70% (*P* = 0.0056) reduced carnosine content in the OB (Fig. 3C). To our knowledge, this is the first demonstration of carnosine measurement in intact brain tissue, also establishing MAS NMR spectroscopy as a fast and convenient way to determine carnosine in this tissue. Furthermore, we performed brain magnetic resonance imaging (MRI) on 94-week-old female *Gadl1*^+/+^, *Gadl1*^+/−^, and *Gadl1*^−/−^ mice. There were no significant differences in the brain morphology of *Gadl1* genotypes (Fig. 3, E to F).

### Substrate specificity of GADL1

Many PLP-dependent enzymes have multiple substrates (*31*). To explore whether the divergent metabolic changes observed in *Gadl1*^−/−^ mice could be secondary to changes in β-alanine levels or were due to the parallel chemical conversion of multiple substrates by GADL1, we solved the crystal structure of mouse GADL1 (*25*). The structure of the closely related CSAD has been solved earlier but not published [Protein Data Bank (PDB) entry 2JIS]. With available crystal structures of GADL1, CSAD, and GAD (*32*), it is possible to identify determinants of substrate specificity in these acidic amino acid decarboxylases. GADL1 and CSAD have different, although slightly overlapping, physiological functions (*24*). GADL1, through the conversion of Asp to β-alanine, is important in carnosine peptide biosynthesis, while CSAD catalyzes the major pathway in taurine synthesis, using CSA as substrate. Despite similar chemical structures of the two substrates (Fig. 3G), these two highly similar enzymes can distinguish between them. Both Asp and CSA are smaller than Glu (Fig. 3H), explaining the inability of GADL1 and CSAD to function as glutamate decarboxylases (Fig. 3I). The carboxyl group in Asp is planar, while the sulfinic acid moiety of CSA is tetrahedral and has slightly longer bonds; effectively, CSA is slightly larger and can, perhaps, more specifically accommodate to a binding site due to its shape and charge.

As the chemical reaction catalyzed by the enzymes is identical, we can assume that the reactive groups bind in an identical manner, and the differences lie in recognizing the side chain of the substrate. GAD uses Glu as a substrate, while GADL1 acts on Asp, indicating that side-chain length is one determinant of productive binding. For binding a negatively charged side chain in the specificity pocket, one expects to find positive charge potential in the form of amino groups. For GAD, these interactions can be conceived; two backbone NH groups and a water molecule coordinated by a conserved His residue coordinate the side-chain carboxyl group in the model of the substrate complex, together with a Ser side chain. How is GADL1 different? The major difference is the substitution of this Ser with a Tyr residue (Fig. 3I), effectively making the binding cavity smaller. Interactions with the peptide backbone and the His-water unit are likely conserved for Asp binding to GADL1. The specificity pocket contains a Tyr (GADL1) or Phe (CSAD) residue, and the slightly different conformations of these residues are enough to cause this difference in substrate specificity.

### Tissue-specific effects of GADL1 on the synthesis of taurine and its derivatives

On the basis of the ability of purified GADL1 to produce hypotaurine from CSA, it has been suggested that the primary biological function of GADL1 may be in taurine synthesis (*23*). However, the concentrations of the substrates and products in taurine synthesis were unaltered in the *Gadl1*^−/−^ mice in the brain, OB, and liver. In contrast, levels of taurine derivatives were moderately reduced in SKM in *Gadl1*^−/−^ (fig. S4). This is consistent with the observations that taurine mainly is synthesized by other routes, such as by CSAD (*33*), and our previous finding that CSAD is expressed in low amounts in SKM (*24*). In all tissues examined, CSAD protein has been reported to be more abundant than GADL1, and purified CSAD has a 5- to 35-fold higher specificity constant for decarboxylation of CSA than GADL1 (*24*). Moreover, as shown here, the qRT-PCR analysis showed that the levels of *Csad* mRNA were at least 10-fold higher than levels of *Gadl1* and were unaffected by the deletion of *Gadl1* exons (Fig. 2N). Thus, we conclude that in organs with a high content of CSAD, GADL1 has a minor role in taurine synthesis. In the absence of CSAD, levels of taurine in the mouse plasma, liver, and brain decreased by 70 to 90%, with the largest decrease in the liver and the smallest in the brain (*33*). This remaining taurine biosynthetic capacity has been attributed to an alternative biosynthetic route of taurine catalyzed by cysteamine dioxygenase from cysteamine, generated from coenzyme A (*34*). However, our results indicate that GADL1 has a small taurine biosynthetic capacity in vivo that may contribute to the overall production of taurine.

### Oxidative stress markers

**Fig. 4:**
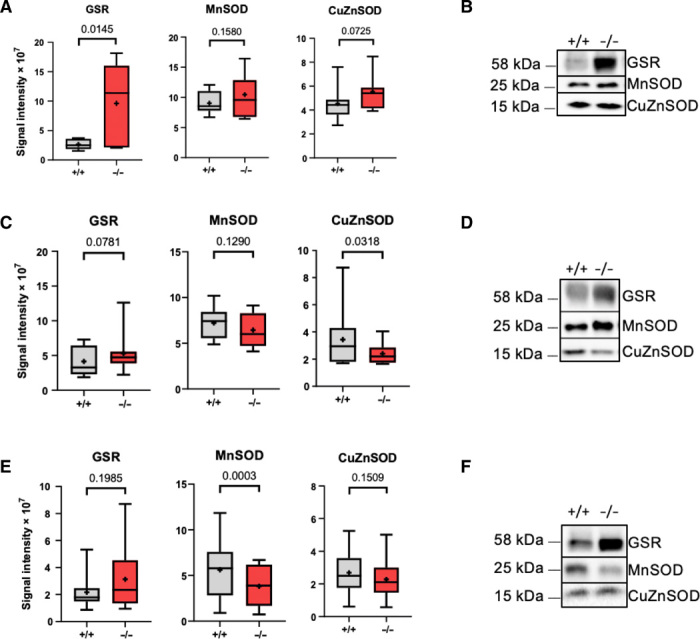
Relative levels of antioxidant enzymes in *Gadl1*^−/−^ mouse tissues. (**A** to **E**) Representative Western blots (B, D, and F) and normalized protein expression levels. (A), (C), and (E) for GSR, SOD1 (CuZnSOD), and SOD2 (Mn) in (A and B) OB, (C and D) cerebral cortex, and (E and F) SKM tissue from female *Gadl1*^+/+^ and *Gadl1*^−/−^ mice.

Several lines of evidence support a role of carnosine peptides in protection against oxidative stress (*1*, *35*). To investigate the role of GADL1 in antioxidant defense, we compared levels of oxidative stress markers in *Gadl1*^+/+^ and *Gadl1*^−/−^ mice. *Gadl1*^−/−^ mice had increased levels of oxidative stress markers, including methionine sulfoxide and γ-glutamyl peptides. These alterations are compatible with elevated glutathione synthesis and consistent with elevated oxidative stress. Some oxidative stress markers were strongly increased even in tissues with a moderate decrease in carnosine peptides, such as the liver. We also compared tissue levels of the antioxidant enzymes superoxide dismutase 1 (SOD1) (CuZnSOD), SOD2 (MnSOD), and glutathione reductase (GSR) in *Gadl1*^+/+^ and *Gadl1*^−/−^ mice (OB, cerebral cortex, and SKM) using Western blotting (Fig. 4). Unexpectedly, the most obvious difference was detected in the OB where the *Gadl1*^−/−^ mice had a threefold increase in GSR levels (*P* = 0.0145) (Fig. 4A).

In contrast to carnosine and anserine, that were reduced in multiple tissues, levels of homocarnosine were significantly increased in the OB of *Gadl1*^−/−^ mice (Fig. 2E). Several other metabolites related to β-alanine synthesis were also altered (fig. S4, G, I, and J).

*Gadl1*^−/−^ mice had also increased levels of many lipid species, including sphingolipids, with the strongest effects in OB (Fig. 2, A to D, and fig. S4K). Notably, complex sphingolipids accumulate under inflammation and oxidative stress, conditions that *Gadl1*^−/−^ mice seem more susceptible to. Moreover, sphingolipids may alter muscle contractility (*36*).

### Animal behavior and tissue morphology

**Fig. 5:**
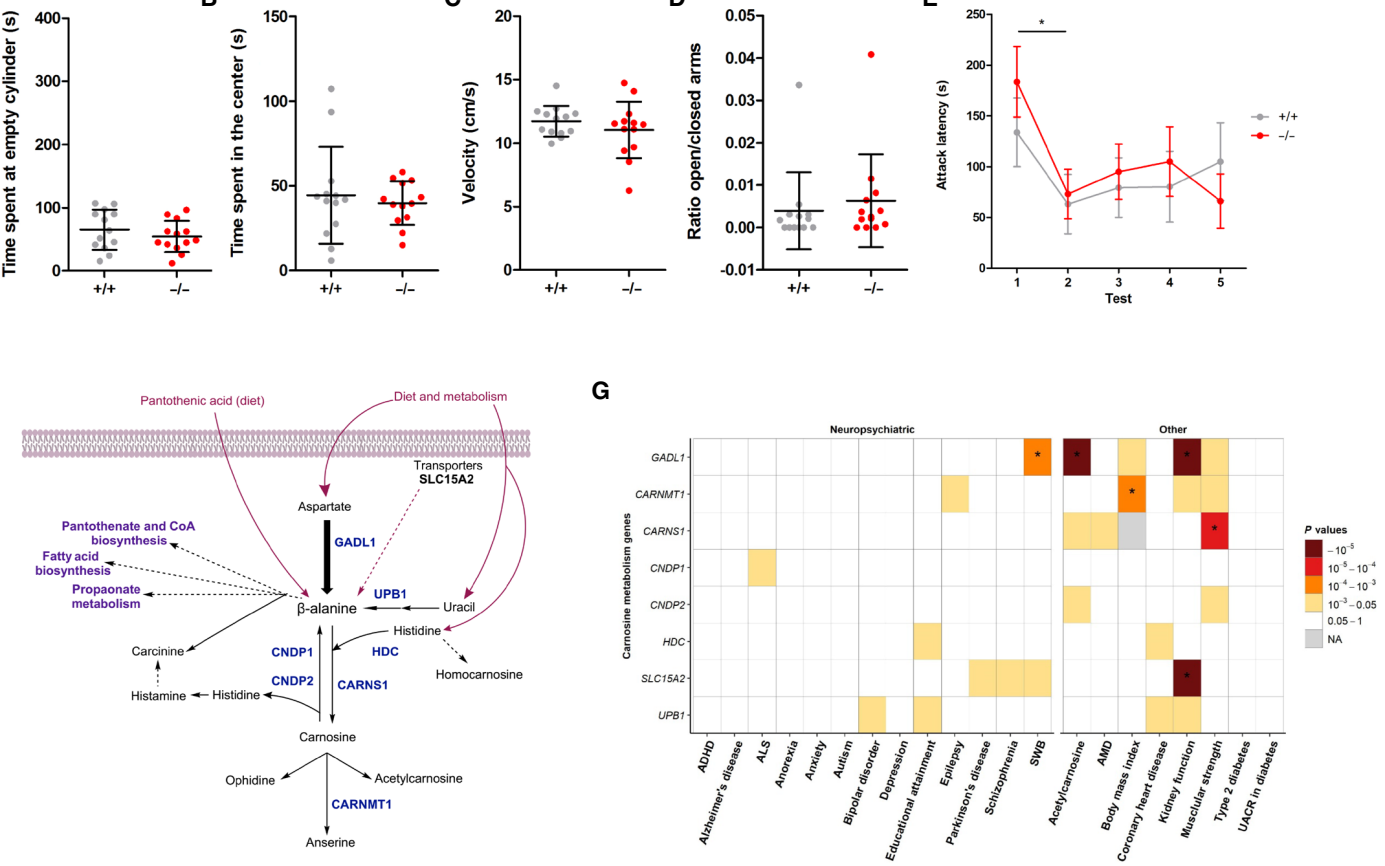
Behavioral phenotypes associated with *Gadl1*^−/−^ mice, carnosine homeostasis and human phenotypes. (**A**) Three-chamber task: Time spent at each cylinder was not different between the genotypes. Both *Gadl1*^+/+^ and *Gadl1*^−/−^ mice prefer the social cylinder, indicating that sociability remains similar. (**B**) Open field task: The cumulative time spent in the center was not different between the genotypes. On this measure, no antianxiety effect was observed. However, on the latency to enter the center (another anxiety measure), *Gadl1*^−/−^ was quicker to enter the center, which may suggest some anxiolytic effects of the *Gadl1*^−/−^ phenotype that would require confirmation in additional studies. (**C**) Open field task: The exploration velocity was not different between the genotypes, indicating that there is no effect on motor function. Similar observations were made with the total distance moved data. Together, this suggests no effect of genotype on activity metrics. (**D**) Elevated plus maze: The ratio of the time spent (s) on the open and closed arms was found not to be different between the genotypes. Both genotypes prefer the closed (sheltered arm), suggesting no difference in anxiety on this measure. (**E**) Resident intruder paradigm: The attack latency against an intruder from the first to the fifth day (tests 1 to 5) was not different between the genotypes. Both genotypes attack faster on the second day compared to the first day after which the attack latency remains constant. This suggests no effect of genotype on aggression. (**F**) A summary overview of different pathways involving β-alanine. Genes analyzed in this study are marked in blue. (**G**) Association analyses of genes involved in the carnosine metabolism. Asterisk indicates statistical significance after correction for multiple testing. ADHD, Attention deficiency hyperactivity disorder; ALS, Amyotrophic lateral sclerosis; SWB, subjective well being; AMD, age-related macular degeneration; UACR, Urine Albumin-to-Creatinine Ratio; NA, not applicable.

To examine the effects of *Gadl1* deletion on brain function, we tested a range of mouse behaviors in *Gadl1*^+/+^ (*n* = 13) and *Gadl1*^−/−^ (*n* = 13) 22-week-old male mice. In the open field, the latency to first enter the center was significantly different between genotypes, with *Gadl1*^−/−^ mice taking less time to enter the center (Fig. 5, A to E). However, the cumulative time spent in the center of the open field was not significant between groups nor was the elevated plus maze and resident intruder test metrics. Only the difference in attack latency between initial testing and retesting 1 day later was significantly different between genotypes. Both genotypes showed a significant preference for the social versus nonsocial cylinder in the three-chamber task (*P* < 0.0001), but there was no significant difference between the genotypes. On this basis, it seems that a reduction of carnosine content in the mouse OB (~70%) and cerebral cortex (~40%) may result in the *Gadl1*^−/−^ mice showing increased initiation to enter an exposed area (which is indicative of decreased anxiety). However, this should be tempered by the observation that the total time spent in the center of the open nor differences in the ratio of the time spent on closed versus open arms of the elevated plus maze were not different. Further investigation of any possible anxiolytic effect of *Gadl1*^−/−^ mice is prudent.

To examine the effects of *Gadl1* deletion on organ aging and morphology, we compared tissue sections from 33 mice aged 28 to 96 weeks, equally matched across different sex and genotypes. At the microscopic level, SKM, brain, and OB morphologies were not affected by the elimination of *Gadl1* (fig. S5A).

### Common genetic variants in the *GADL1* locus are associated with multiple human phenotypes

GWA studies (GWASs) have shown associations of the *GADL1* locus with multiple human traits and diseases, including eating disorders (*37*), kidney functions (*38*), and several blood metabolites. This includes a strong association with levels of acetylcarnosine (*P* = 8.17 × 10^−21^) in the *GADL1* locus at an intergenic SNP rs6804368 (13.9 kb from *GADL1* and 17.9 kb from *TGFBR2*) (*26*). The strongest association (*P* = 5.50 × 10^−37^) has been reported for the response to lithium (Li^+^) in bipolar disorder at an SNP (rs17026688) located in intron 6 of *GADL1* (*21*). However, these findings were not replicated in other clinical samples (*24*).

To get an overview of genetic associations between human phenotypes and enzymes in carnosine metabolism, we conducted gene-based tests using the MAGMA software (*39*), mainly focusing on traits related to oxidative protection and brain functions. We included associations of common variants in *GADL1* and seven related genes (Fig. 5) (*40*). We limited the search to 21 phenotypes with openly available GWAS summary statistics obtained from large samples (total number of tests 167; Bonferroni-corrected *P* value threshold = 2.99 × 10^−4^). Table S3 shows an overview of the data used.

*GADL1* was associated with serum acetylcarnosine levels (*P* = 1.25 × 10^−11^), kidney function (*P* = 1.03 × 10^−7^), and subjective well-being (*P* = 1.22 × 10^−4^). *CARNMT1* was associated with body mass index (*P* = 1.12 × 10^−4^). *CARNS1* was associated with right-hand grip strength (muscular strength) (*P* = 4.98 × 10^−5^). *SLC15A2* was associated with kidney function (*P* = 8.60 × 10^−12^) In addition, several tentative gene-phenotype associations did not pass the Bonferroni-corrected threshold for significance. In conclusion, of eight tested genes known to be involved in carnosine metabolism, *GADL1* and *SLC15A2* showed the strongest association with human phenotypes. The most obvious associations were observed for acetylcarnosine levels, muscle strength, kidney function, and general well-being but not in a range of neuropsychiatric diseases that have been reported to respond to carnosine treatment (Fig. 5, G and F) (*14*).

## DISCUSSION

Carnosine peptides were found in 1900, yet their biological functions and biosynthetic routes are still being debated (*1*). Here, we report the first animal model lacking GADL1. We show that these animals have low levels of β-alanine and carnosine peptides, consistent with a key role of GADL1 in their synthesis. This enzyme is analogous to Asp decarboxylases previously only found in prokaryotes and insects (*23*).

Carnosine peptides, except for homocarnosine, were significantly depleted in the brain and SKM. In contrast, the reduction was modest and nonsignificant in multiple other tissues including the kidney, heart, whole blood, and serum. Furthermore, β-alanine was only reduced significantly in OB. As GADL1 activity was eliminated in *Gadl1*^−/−^ mice, the remaining β-alanine and its peptide derivatives may be derived either from the diet, by gut bacteria, maternally transferred from heterozygous mothers used for breeding, or by de novo synthesis. Biochemical analyses of the standard plant-based rodent diet did confirm low levels of β-alanine and carnosine, but we cannot exclude a possible small dietary contribution from β-alanine, pantothenic acid (vitamin B5), or carnosine peptides (table S4). It is, however, more likely that there are several alternative biosynthetic routes for β-alanine and that these have different roles in different tissues. Thus, in tissues with the highest demands for carnosine synthesis, i.e., SKM, brain, and particularly the OB, we demonstrate a critical role of de novo β-alanine biosynthesis to maintain its levels, as well as compensatory up-regulation of enzymes involved in alternative biosynthetic routes. As blood levels of β-alanine and carnosine peptides were moderately affected by elimination of GADL1, it seems that the muscle and brain are less dependent on uptake of these compounds from the circulation.

Alternative biosynthetic routes may include β-alanine synthesis by CSAD, which has a relatively low β-alanine synthetic specific activity but much higher tissue abundance than GADL1 (*24*), production of β-alanine from uracil by ureidopropionase 1 [β-alanine synthase, UPB1; *N*-carbamoyl-β-alanine amidohydrolase; (BUP1/UPB1)] (*41*), or by other enzymes, such as β-alanine-2-oxoglutarate transaminase (ABAT) or β-alanine pyruvate transaminase (AGXT2) (*20*). Our findings contrast earlier observations that found no decarboxylation of Asp in rat muscle (*23*). Dietary PLP supplementation significantly increases the concentrations of β-alanine and carnosine peptides in the SKM of rats (*20*). As GADL1 is dependent on PLP, this increase may be due to the activation of GADL1 by PLP. In contrast, PLP is not required for the production of β-alanine by UPB1. Our observation of increased *Upb1* transcript levels in the OB of *Gadl1*^−/−^ mice but unaltered levels of *Abat* and *Agxt2* indicates a role UPB1 in maintaining levels of β-alanine. However, this finding contradicts earlier observations of UPB1 protein in the rat liver and kidney but not in the brain, lung, SKM, or spleen (*41*).

In contrast to the reduced levels of β-alanine, carnosine, anserine, and acetylcarnosine, the levels of homocarnosine were increased. This is probably the result of a shortage of β-alanine precursor and carnosine peptides, indicating that the tissue levels of these dipeptides may be tightly regulated. Moreover, no significant difference in GABA between *Gadl1*^+/+^ and *Gadl1*^−/−^ mice was detected. Together with the observations of increased homocarnosine (a GABA-derived peptide), this indicates that GADL1, despite its name, does not catalyze the decarboxylation of glutamate to GABA. Figure 5F summarizes the different biosynthetic routes of carnosine peptides, and the main enzymes and transporters believed to be involved in this metabolism in mammalian tissues.

As the *Gadl1* locus on chromosome 3p24.1-3p23 is close to the *Tgfbr2* locus (3p24.1) and genetic variants in the vicinity of *Gadl1* influence plasma levels of transforming growth factor–β (TGF-β) receptor type 2 (TGFBR2), the gene-targeting strategy was designed to avoid regulatory sequences in this region. This strategy resulted in the expression of low amounts of a catalytically inactive and unstable partially truncated version of the GADL1 protein, but *Tgfbr2* transcripts were unaltered.

To determine the substrate specificity of GADL1, we previously expressed the mouse enzyme in bacteria and screened purified GADL1 against all proteinogenic amino acids, as well as many other putative substrates and inhibitors, demonstrating decarboxylase activity against Asp, CSA, and cysteine (*24*). This substrate profile was similar to what Liu *et al.* (*23*) observed for the human enzyme. However, compared to other PLP-dependent decarboxylases, the affinity and selectivity were very low for all substrates tested. Liu *et al.* (*23*) reported that it was impossible to detect GADL1 enzyme activities in tissue lysates. Here, we show that, despite its extremely low activity in vitro, GADL1 is important for β-alanine and carnosine peptide synthesis, particularly in the OB, cerebral cortex, and SKM.

*Gadl1*^−/−^ mice also had decreased levels of taurine and multiple taurine derivatives in SKM, indicating that CSA also is a physiologically relevant substrate. *Gadl1*^−/−^ mice also showed other biochemical alterations that could be secondary to the depletion of β-alanine derivatives and antioxidant function or other effects of GADL1 inactivation. *Gadl1* deletion might alter energy metabolism through several pathways. In addition to its role in carnosine synthesis, β-alanine is a component in pantothenic acid (vitamin B5) and coenzyme A, an essential cofactor for multiple biochemical pathways, including energy metabolism.

The role of GADL1 as a relatively nonspecific decarboxylase of low molecular weight acid substrates is consistent with its relatively high Michaelis-Menten constant (*K*_m_) values and low catalytic efficacy (*24*). Mammalian genomes encode hundreds of PLP-dependent enzymes, many of which may catalyze new or unclassified reactions (*31*). In addition, because of their common mechanistic features and similar structures, many PLP-dependent enzymes can catalyze multiple biochemical reactions. This promiscuity makes it difficult to define their primary biological functions (*31*). Some enzymes have evolved to have multiple physiological substrates. Such a reactivity with multiple substrates may be advantageous for fitness (*42*). GADL1 may be an example of an enzyme with multiple biological activities. Although the strongest effect of GADL1 deletion seemed to be a decrease in β-alanine and carnosine peptides, with the taurine pathway being intact, the *Gadl1*^−/−^ mice also had slightly reduced levels of taurine derivatives in SKM, consistent with multiple catalytic functions of this enzyme. Similarly, while the accumulation of oxidative stress biomarkers in *Gadl1*^−/−^ mice is probably secondary to loss of β-alanine or carnosine peptides, we cannot exclude that the metabolic changes could be caused by other functions of GADL1.

Inspection of the catalytic site of mouse GADL1 shows that it might be accessible by different small, acidic, polar substrates. From the comparison (Fig. 3I), it can be observed that very minor changes in specificity-determining amino acids in the active site vicinity may dictate the function of an enzyme at the tissue and organism level. These structural features correspond well with the previously reported substrate specificity of purified mouse and human GADL1 (*23*–*25*). For a more detailed understanding of the catalytic properties of GADL1, as well as its closest homologs, high-resolution structural studies with active-site ligands, preferably with the flexible catalytic loop visible, will be required.

Both in vitro and in vivo experiments have shown that reactive oxygen species can decrease the levels of antioxidant enzymes, such as SOD and glutathione peroxidase, and that carnosine supplementation can restore depleted levels of these enzymes (*1*). Carnosine peptides are considered to be important in protection, e.g., against ischemia-related free radical damage (*1*). However, the protective function of carnosine has only been studied in vitro or in animals receiving large pharmacological doses of carnosine. Thus, β-alanine and carnosine dietary supplementation have widespread human use and are marketed to protect against oxidative stress (*1*). However, contradictory findings have been reported, and elevated levels of β-alanine were noted to reduce cellular taurine levels and to be associated with increased oxidative stress (*43*) and altered energy metabolism (*44*). Here, we show that the *Gadl1* deletion mice have increased levels of oxidative stress biomarkers and altered levels of several antioxidant enzymes. These findings establish this mouse model as a new tool to study not only β-alanine synthesis but also carnosine peptide biology and their relationship to oxidative stress and diseases.

Deletion of the mouse carnosine transporting dipeptide transporter *Pept2 (SLC15A)* has previously been reported to alter carnosine levels in several organs but not as markedly as in the *Gadl1*^−/−^ mice (*45*). These organ-specific effects indicate that although β-alanine and carnosine peptides are mainly synthesized locally in the OB, other organs mainly take up carnosine peptides and their precursors from the circulation. Carnosine homeostasis in SKM is regulated by multiple nutritional and hormonal stimuli in a complex interplay between transporters and enzymes, including *CARNS*, *CNDP2*, *PHT1*, *PHT2*, *TauT*, *PAT1*, *ABAT*, and *HDC* (Fig. 5F) (*40*). It was recently reported that in the SKM, *CARNS1* and *GADL1* are rapidly up-regulated upon high-intensity exercise, demonstrating the importance of carnosine in muscle function and physiological regulation of these enzymes (*46*). The importance of GADL1 in carnosine synthesis is supported by human genetic studies showing a strong association of *GADL1* variants with blood levels of carnosine peptides (*26*). However, the tissue levels of carnosine peptides are reflecting levels of not only β-alanine and histidine and their biosynthetic enzymes but also other metabolites and enzymes. Thus, the enzymes and transporters involved in carnosine homeostasis are nonspecific and subjected to competitive inhibition by alternative substrates, which may differ between tissues (Fig. 5) (*47*).

Since *Gadl1*^−/−^ mice displayed behavioral changes and β-alanine and carnosine peptides have been implicated in many different physiological functions and disease states, we performed a gene-based analysis of *GADL1* and enzymes and transporters directly involved in carnosine metabolism. We selected 21 human diseases and traits related to oxidative stress and where treatment with carnosine peptides has shown some promising effects (Fig. 5G) (*12*, *48*). In addition to the strong associations with plasma carnosine peptide levels, common variants in the *GADL1* locus were associated with subjective well-being and muscle strength. The association with subjective well-being is particularly intriguing. This phenotype is genetically related to somatic complaints not only as bodily aches and pains but also as low energy, anxiety, and depression, traits that have all been shown in animal studies to be related to levels of carnosine peptides in the brain and SKM (*49*). However, for most of the diseases studied, we observed no significant associations with genes associated with carnosine homeostasis. Still, we cannot exclude possible effects of rare genetic variants or a role in other patient groups or populations. The behavioral data in the *Gadl1*^−/−^ mice suggest that the link between *GADL1* with anxiety is worth exploring further, including the investigation of differences in sensory processing and alterations in fear-related behavior.

Brain metabolomics has revealed large regional differences, with particularly large concentration gradients for carnosine (*50*). However, it is still a mystery why GADL1 and its carnosine peptide products are so abundant in OB. In zebrafish, high immunoreactivity for carnosine and anserine is found in sensory neurons and nonsensory cells of the olfactory epithelium, olfactory nerve, and the OB, indicating a specific role of these peptides in sensory organs (*51*). On the basis of the early occurrence of carnosine peptides during embryonic development, it has been suggested that these peptides play a role in the developing nervous system, specifically in the olfactory and visual function (*51*). Our findings of intact histological architecture and the modest behavioral effects of *Gadl1* deletion may argue against this hypothesis. However, it is also possible that the remaining 10 to 30% carnosine peptides are adequate for maintaining these biological effects, as reported for other neurotrophic factors such as serotonin (*52*). Alternatively, a neuroprotective, antioxidant role of GADL1 and carnosine peptides is conceivable. The olfactory epithelium provides a direct entry route from the environment to the OB and brain for substances that could cause oxidative stress. These substances can cause age-related cellular degeneration and olfactory damage, and prevention of this damage depends on an intact antioxidant defense system. Although baseline GADL1 levels are low, the levels of this enzyme may be up-regulated in response to oxidative damage and other physiological stressors, as shown in oligodendrocytes obtained from a mouse model of amyotrophic lateral sclerosis (*53*) and in SKM upon exercise (*46*).

## CONCLUSIONS

GADL1 is essential for the production of β-alanine and carnosine peptides, particularly in the brain and SKM. Mice lacking GADL1 have behavioral changes, increased oxidative stress markers, and age-related changes, underscoring the biological importance of these peptides. Common variants in the human *GADL1* locus are associated with plasma levels of acetylcarnosine, muscle strength, kidney function, and subjective well-being. Future studies should explore how this enzyme is regulated and its involvement in physiological functions, including antioxidant defense mechanisms, and pathological states.

## MATERIALS

Chemicals in this study were purchased from Sigma-Aldrich if not otherwise stated. Probes for the qRT-PCR were from Thermo Fisher Scientific, the kits used were from Thermo Fisher Scientific or QIAGEN, agarose was from Apollo Scientific, and SDS–polyacrylamide gel electrophoresis (PAGE) gels were from Bio-Rad. *O*-Phthaldialdehyde reagent and 2-mercaptoethanol were from Merck Schuchardt. l-Carnosine nitrate salt was used as standard for NMR spectroscopy.

## METHODS

### Ethical statement

This study was carried out per Norwegian laws and regulations and the European Convention for the Protection of Vertebrate Animals used for Experimental and Other Scientific Purposes. The protocol was approved by the Animal Studies Committee, Norwegian Food Safety Authority (Mattilsynet, permit number 9468).

### Generation of *Gadl1*-null mice

*Gadl1*-null (−) mice were generated by homologous recombination in C57BL/6N mice at genOway, Lyon, France. On the basis of the *Gadl1* complementary DNA (cDNA) sequence NM_028638, the exon/intron organization of the gene was established. The mouse *Gadl1* gene is located on chromosome 9 and extends over more than 183.6 kilo–base pairs (kbp) (sequence map Chr9:115909455-116074347 bp, +strand). The mouse *Gadl1* locus consists of 20 exons (Fig. 1). ATG initiation codons are located in exons 3, and stop codons are located in exons 19 and 20. Bioinformatic analysis identified three isoforms for GADL1 protein and three predicted other splice variants of the *Gadl1* gene. In the National Center for Biotechnology Information database release 106, four isoforms with predicted 518, 479, 362, and 502 amino acid residues (NM_028638) were identified. On the basis of sequence comparison and x-ray structural data (*25*), Lys^405^, Lys^333^, and His^219^ are predicted to be essential amino acids of the PLP-binding region, and Arg^494^ and Gln^120^ are essential amino acids of the substrate-binding region. Exon 7 is highly conserved and contains the substrate-binding residue Gln^120^. This exon is present in all splice variants. In the mouse genome, the closest coding gene is *Tgfbr2*, encoding the TGFBR2 (sequence map Chr9:116087695-116175363 bp, −strand). This is involved in multiple biological pathways and cells. In addition, several noncoding genes and pseudogenes are located in this region. To minimize the risk of interfering with possible regulatory sequences, it was decided to delete only exon 7 in the *Gadl1* gene, resulting in the deletion of sequences encoding part of the active site domain of *Gadl1*, including Q120 (*24*). The mouse model was generated by homologous recombination in embryonic stem (ES) cells. For this purpose, a targeting vector containing regions homologous to the genomic *Gadl1* sequences was constructed. After its transfection into C57BL/6N ES cells, the recombined cell clones were injected into blastocysts. Mouse breeding was established with C57BL/6N Flp-deleter mice to remove the neomycin cassette. The heterozygous KO colony was produced by breeding with C57BL/6N Cre-deleter mice.

### Breeding

Heterozygous siblings were mated to produce *Gadl1*^−/−^ homozygous pups. Further breeding and genotyping were performed at the animal facility at the University of Bergen (Bergen, Norway). Animals were group-housed in temperature- and light-controlled vivarium (21° ± 1°C; 12:12-hour light/dark artificial circadian rhythm). All experiments were conducted with 3- to 96-week-old mice of both sexes that were ad libitum fed using standard rodent diets [rat and mouse no. 1 (RM1)] during maintenance and RM3 during breeding (Scanbur/Special Diets Services, Witham, UK). These diets are plant-based. The content of natural amino acids is specified, but the content of carnosine peptides is not specified (http://sdsdiets.com/pdfs/RM1-A-P.pdf). Further analysis is shown in table S4.

### *Gadl1* mouse genotype determination

DNA was extracted from mice ear samples at 2 weeks of age. Genotyping was performed using a multiplex PCR kit (#206143, QIAGEN). After initial heat activation at 94°C for 15 min, DNA was denatured at 94°C for 30 s, annealed at 60°C for 1.30 min, and extended at 72°C for 1.30 min. This cycle was repeated 25 times. DNA was extended for an additional 30 min after the last cycle. PCR products were analyzed using 2% agarose gel electrophoresis. The PCR product size was measured to 166 bp in the *Gadl1*^−/−^ mice and 330 and 750 bp in the *Gadl1*^+/+^ mice. All three base pair sizes were measured in the *Gadl1*^+/−^ mice (166, 330, and 750 bp). The sizes of PCR products and the primer sequences are summarized in table S5 and Fig. 1E.

### Validation of the animal model

The deletion strategy was validated using DNA sequencing, RNA sequencing, proteomic analyses, and Western blotting. DNA sequencing confirmed that exon 7 was absent from the genomic DNA of the *Gadl1*^−/−^ mice. We determined branching scores that reflected each exon’s splicing capability using the RNA splicing prediction tool SROOGLE (*54*). This gave a lower splicing score for exon 8 compared to exon 9, suggesting that splicing of exon 6 to exon 9 was favored over splicing to exon 8. The qRT-PCR data also suggested that the *Gadl1* mRNA from *Gadl1*^−/−^ mice was stable. However, sequence analysis showed that both exons 7 and 8 were absent from *Gadl1* mRNA in *Gadl1*^−/−^ mice. An mRNA devoid of exons 7 and 8 is expected to be stable and produce a truncated protein. Expression and purification of mouse GADL1 protein in *E. coli* were performed as described (fig. S2) (*24*). Site-directed mutagenesis of mouse GADL1 to generate a protein lacking exons 7 and 8 (coding protein sequence, NHPRFFNQLYAGLDYYSLAARIITEALNPSIYTYEVSPVFLLVEEAVLKKMIECVGWKEGDGIFNP) was performed using the following primers: GADL1, 5′-TTCAGAACCGCCTCTTCCAC-3′ (reverse), 5′-TCCAAGATTTTTCAACCAGC-3′ (foward), 5′-AGAAAGCACCGCCGGCTCCT-3′ (forward), 5′-GGATGAGATAGACAGCCTGG-3′ (forward), 5′-TTTCTGTTCATGGGGGTAAT-3′ (forward), 5′-TGCGGTATTCGGAATCTTGC-3′ (reverse), 5′-ACGCATCGTGGCCGGCATCA-3′ (reverse), 5′-CGATTTCGGCCTATTGGTTA-3′ (forward), 5′-TGCACAATCTTCTCGCGCAA-3′ (reverse), 5′-ATGGGGGATCATGTAACTCG-3′ (forward), 5′-CTTGCTGCAACTCTCTCAGG-3′ (reverse), 5′-CGGATCAAGAGCTACCAACT-3′ (forward), 5′-TAACGAAGCGCTGGCATTGA-3′ (reverse), and 5′-GCCTTTGAGTGAGCTGATAC-3′ (forward).

### Metabolomic studies using LC-MS

Animals were anesthetized using urethane (250 mg/ml) administered by intraperitoneal injection and adjusted to their individual weight (1.4 to 1.8 mg/kg). Tissues were perfused with 50 ml of 0.02% heparin-saline solution pumped through the heart’s left ventricle before organ extraction. After dissection, the tissues were flash-frozen in liquid nitrogen and stored at −80°C until use. A total of 172 different tissue samples from 41 animals were shipped at −80°C to Metabolon Inc. (Durham, NC, USA) for further processing. Male and female mice were matched according to genotype and age. Table 2 shows the list of mice used in the metabolomic study.

All 41 animals were analyzed for brain, SKM, and liver metabolites. A subset of 21 animals (10 *Gadl1*^+/+^ *and 11 Gadl1*^−/−^, 4.63 and 4.89 weeks, respectively) were analyzed for OB metabolites. Exploratory analyses were performed in the kidney, cerebellum, heart, whole blood, and serum from 12-week-old males (seven *Gadl1*^+/+^ and seven *Gadl1*^−/−^). Because of limited capacity of the animal breeding facility, samples were dissected and shipped in three separate batches, with tissues from 10 (5 *Gadl1*^+/+^ and 5 *Gadl1*^−/−^*)*, 17 (8 *Gadl1*^+/+^ and 9 *Gadl1*^−/−^*)*, and 14 (7 *Gadl1*^+/+^ and 7 *Gadl1*^−/−^*)* animals, respectively. All samples were processed using an identical pipeline.

At the Metabolon Company, samples were prepared using the automated Microlab STAR® system from the Hamilton Company. To remove protein, dissociate small molecules bound to protein or trapped in the precipitated protein matrix, and to recover chemically diverse metabolites, proteins were precipitated with methanol under vigorous shaking for 2 min (Glen Mills Geno/Grinder 2000), followed by centrifugation. The resulting extract was divided into four fractions for analysis using different chromatographic methods. Each sample extract was stored overnight under nitrogen before preparation for analysis. After that, the samples were reconstituted in appropriate solvents (according to the chromatographic method) contained a series of standards at fixed concentrations to ensure injection and chromatographic consistency.

Metabolomic analyses were performed through three chromatographic methods used that were based on reversed-phase/ultraperformance liquid chromatography (UPLC)-MS/MS chromatography with positive and negative ion mode electrospray ionization (ESI) and one chromatographic method using hydrophilic interaction liquid chromatography/UPLC-MS/MS with negative ion mode ESI. The columns used were Waters UPLC BEH C18 (2.1 mm by 100 mm, 1.7 μm) and Waters UPLC BEH Amide (2.1 mm by 150 mm, 1.7 μm).

Metabolomic analyses were performed at UPLC (Waters ACQUITY) coupled to Thermo Fisher Scientific Q Exactive high-resolution/accurate mass spectrometer interfaced with a heated ESI (HESI-II) source and Orbitrap mass analyzer operated at 35,000 mass resolution. The MS analysis alternated between MS and MS^n^ scans using dynamic exclusion. The scan range varied slighted between methods but covered 70 to 1000 mass/charge ratio (*m/z*).

Compounds were identified by comparison to library entries of purified standards or recurrent unknown entities. Metabolon maintains a library based on authenticated standards that contain the retention time/index (RI), *m/z*, and chromatographic data (including MS/MS spectral data) on all molecules present in the library. Furthermore, biochemical identifications are based on three criteria: retention index within a narrow RI window of the proposed identification, accurate mass match to the library ± 10 parts per million, and the MS/MS forward and reverse scores between the experimental data and authentic standards. The MS/MS scores are based on a comparison of the ions present in the experimental spectrum to the ions present in the library spectrum.

Normalized areas of each compound were rescaled to set the median equal to 1. The values obtained for each tissue were used for metabolomic analyses with MetaboAnalyst 4.0. Analyses of PLS-DA data were normalized using Pareto scaling.

#### ^1^H-NMR spectroscopy

MAS ^1^H-NMR spectroscopy was performed using samples (13 to 20 mg of wet weight) of the OB from 12-week-old male mice of different genotypes *Gadl1*^−/−^*, Gadl1*^+/−^, and *Gadl1*^+/+^. After dissection, the samples were stored at −80°C before they were placed in 4-mm ZrO_2_ MAS rotors with 50 μl of inserts and analyzed in a 500-MHz Bruker instrument at 4°C. Water signal decoupling was achieved by presaturation pulses. Each spectrum was recorded at an MAS rate of 5 kHz using 256 transients and a 5-s delay between each transient. To aid comparison, the maximal signal intensities were normalized to the same level for all samples.

#### ^1^H-MRI of living animals

Animals were anesthetized using 5 to 6% (v/v) sevoflurane (SevoFlo, Zoetis Inc., Kalamazoo, MI, USA) mixed with oxygen (200 cm^3^/min) for induction and ~3% for maintenance. During the MRI experiments, the respiration rate was monitored using a pressure sensor (SA Instruments, NY, USA). MRI investigations were performed with User PharmaScan 70/16 (7 T) scanner from Bruker (Bruker BioSpin GmbH, Ettlingen, Germany) using a 72-mm–inner diameter transmit coil together with a mouse brain four-element surface coil array for receiving the MR signal. We recorded 13 coronal T2-weighted images [slice thickness, 0.5 mm; slice distance, 0.05 mm; field of view (FOV), 20 mm by 20 mm; matrix size, 256 by 256] with fat suppression. The images were recorded with a turbo-RARE sequence [time to echo (TE), 38 ms; repetition time (TR), 3200 ms; four averages]. The FOV covered the whole brain including the OB. The software used with the scanner was ParaVision 6.0.1. Analysis of MR images was performed with Fiji ImageJ (version 1.46a, National Institute of Health, Bethesda, MD, USA). The OB was manually delineated in each image slice, and the number of pixels within each region of interest was translated to volume measurement.

#### RNA purification and qRT-PCR

Total RNA was purified from different tissues from both *Gadl1*^+/+^ and *Gadl1*^−/−^ mice using the RNeasy purification kit from QIAGEN (#74104; and for muscle tissue, #74704). cDNA was synthesized in triplicates from this RNA using a High-Capacity RNA-to-cDNA kit (#4387406, Applied Biosystems). qRT-PCR was performed using the TaqMan gene expression assay (TaqMan Gene Expression Master Mix, #4369016, Applied Biosystems). The TaqMan probes were as follows: Mm00520087_m1 (CSAD), Mm01348767_m1 (GADL1, exons 13 to 14), Mm01341531_m1 (GADL1, exons 3 to 4), Mm01341534_m1 (GADL1, exons 6 to 7), Mm99999915_g1 [glyceraldehyde-3-phosphate dehydrogenase (GAPDH)], and Mm00607939_s1 (β-actin). mRNA expression for all genotypes was normalized against the housekeeping genes GAPDH and β-actin using a variant (2^Δ*C*t^) of the Livak method (2^−ΔΔ*C*t^), as described in the real-time PCR applications guide from Bio-Rad (*55*). Values are presented as means and upper 95% limit on an ln *y* scale.

#### RNA sequencing

RNA sequencing was performed using the TruSeq stranded mRNA prep kit from Illumina and an Illumina HiSeq 4000 instrument (paired end, 75 bp, ×2 run). Raw data were quality controlled using the FastQC tool (available at www.bioinformatics.babraham.ac.uk/projects/fastqc). Raw reads were aligned to mouse genome M13 using HISAT2 (*56*). Read aligned to coding regions of the genome were counted using “FeatureCounts” (*57*). Normalization and differential gene expression were performed using DESeq2 (*58*). Transcripts from 27,878 genes at an average were detected. GO enrichment analysis was performed using tools “TopGo” and their dependencies in R environment (version 2.36.0). Biological pathways affected by the *Gadl1* deletion were defined according to GO (*27*, *28*) and the KEGG (*27*, *59*). Among the 27,878 mouse genes reliably identified, we further analyzed up- or down-regulated genes with a log_2_ fold change either less than −1 or greater than +1 and *P* ≤ 0.05. Heat maps were made using ClustVis (*59*).

#### Western blotting

We used custom-made affinity-purified sheep antibodies generated against purified human GADL1 MBP (maltose-binding protein) fusion proteins (J. Hastie, University of Dundee). All the other antibodies were purchased from Abcam. The primary antibodies were anti-SOD1 (1:2000; CuZn; ab13498), anti-SOD2 (1:1000; Mn; ab68155), and anti-GLR1/GSR (1:5000; ab16801) prepared in 3% (w/v) bovine serum albumin (BSA) in 1× tris-buffered saline (20 mM Trizma base and 150 mM NaCl) with 0.1% (v/v) Tween 20 (TBST). Frozen tissue samples (OB, SKM, and cerebral cortex) were homogenized in radioimmunoprecipitation assay buffer containing a protease inhibitor cocktail. Equal amounts of protein (30 mg) were separated by SDS-PAGE and transferred to a nitrocellulose membrane (Bio-Rad) using a Trans-Blot Turbo transfer system (Bio-Rad) according to the manufacturer’s protocols. Membranes were blocked with 3% BSA in TBST for 2 hours at room temperature before incubation with primary antibodies overnight at 4°C. The following day, membranes were incubated with the secondary antibody, anti-rabbit and goat anti-sheep horseradish peroxidase immunoglobulin G H + L (1:10,000; ab6721) (Bio-Rad, Hercules, CA), and developed with the enhanced chemiluminescence technique using a WesternBright Sirius kit (#K-12043-D20, Advansta) on a Gel DocTM XR+ system (Bio-Rad) with Image Lab software (version 6.0.1). All blots were normalized against the total protein load in a *Gadl1*^+/+^ mouse.

#### Behavioral tests

There were 26 male mice, 13 mice from each genotype (*Gadl1*^+/+^ and *Gadl1*^−/−^) tested for the behavioral experiments. The average age of *Gadl1*^+/+^ and *Gadl1*^−/−^ mice was 22.2 and 21.7 weeks, respectively. The order of testing of the mice was randomized for each test with the tasks following the order, three-chamber social interaction task, open field, elevated plus maze, and resident intruder task. Exclusively male mice were used to eliminate possible variation caused by the estrous cycle of female mice. Upon entry, all mice were provided with a unique tail number. All mice were housed at the institutional animal facility in an individually ventilated cage (type 2L, Tecniplast S.p.A., Buguggiate, Italy) with an igloo as environmental enrichment and had ad libitum access to water and food. Mice were socially isolated before the open field task. The mice were housed under a reversed light/dark cycle (12:12 hours) in a ventilated cabinet, Scantainer (Scanbur, Karlslunde, Denmark), with sunset at 7:30 a.m. at a constant temperature of 24° ± 1°C. All experimental procedures were approved by the Committee of Animal Experiments of the Radboud University Medical Center (project number DEC2016-0094), Nijmegen, The Netherlands.

All the behavioral experiments were performed in the dark phase under red light conditions in the same experimental room. No experiments were performed within the first hour after the light/dark transition. All animals were given 1 month to acclimatize to the animal housing facility before behavioral testing. EthoVision XT9 software (Noldus, Wageningen, The Netherlands) was used for tracking the mice and analysis of the videos in the open field and elevated plus maze experiments with the Observer version 11 software (Noldus, Wageningen, The Netherlands) used for the assessment of the social interaction and resident intruder task experiments. All the behavioral tests were recorded using a high-speed (25 frames/s) infrared camera (GigE, Basler AG, Ahrensburg, Germany). MediaRecorder (Noldus, Wageningen, The Netherlands) was used to record these movies.

#### Open field

Locomotion activity was quantified in a 55 cm by 55 cm by 36 cm activity chamber that was positioned on a flat table with a camera directly above the center of the apparatus. The animals were placed in the center of the field where locomotion activity was then recorded for 5 min. The arena was divided into four quadrants in which the connected center points of all quadrants formed the center of the field and measured 27.5 cm by 27.5 cm. The total time in the center zone, outside the center, and time spent near the walls was measured, as well as the frequency of center visits. In addition, the latency to leave the center was used as an indication of immobility behavior. Reduced frequency of center visits, velocity, and distance traveled was used as indications of locomotor activity and anxiety behavior. Total distance traveled was measured by tracking movement from the center of the mice’s body. The arena was cleaned with 70% alcohol between tests. Videotapes of the locomotion activity were examined using EthoVision XT9 (Noldus, Wageningen, The Netherlands).

#### Elevated plus maze

The apparatus provided by Noldus consisted of two open arms and two closed arms (36 cm by 6 cm, 15-cm-high walls) with a common central platform (6 cm^2^). The apparatus was placed 60 cm above the ground. At the start, animals were placed at the junction of the open and closed arms with the head facing the closed arms. The elevated plus maze was performed in the dark phase under dim light conditions. The time spent in the open and closed arms were examined as a time ratio (RT). The RT is the time spent in the open arms (TO) divided by total time spent in both closed (TC) and open arms (TO): RT = TO/(TO + TC). Furthermore, the frequency of transition between the arms, the total distance traveled, and velocity was measured. Between each animal, the maze was cleaned with 70% ethanol and dried before testing the next animal.

#### Resident intruder paradigm

The resident intruder paradigm was performed to assess territorial aggression. On each testing day, an unfamiliar C57BL/6J intruder mouse was encountered, which was randomly assigned to a resident for each interaction. All animals, both resident and intruder, were tested once a day. The housing cage of the resident was used as the interaction area. A transparent Plexiglas screen was placed in the middle of the cage to prevent direct interaction between animals but to enable visual, auditory, and olfactory perception. The intruder mouse was placed at the other side of the plastic screen for a period of 5 min. Hereafter, the screen was removed, and the interaction was videotaped for 5 min. After the test, the intruder was removed from the cage, and both animals were weighed and checked for wounds. The frequency of attacks and bites and the latency to the first attack were analyzed manually for all interaction days. An attack latency of 300 s was taken in case no attack occurred within the 5-min interaction window.

#### Three-chamber social interaction test

A standard three-chamber task arena (Noldus, Wageningen, The Netherlands) was used in a dark room with red overhead lighting. During the first 10 min of the test, the test mouse was placed alone in the arena to habituate to the new environment. In both the left and right chambers, an empty acrylic cylinder with bars was placed. After 10 min, an interaction mouse (C57BL/6J mouse, same age as test mouse) was placed in a cylinder, randomly in either the left or right cylinder. Testing was spread across 2 days with each C57BL/6J mouse used maximally four times as an interaction mouse. The order of testing was counterbalanced. For 10 min, test mice were allowed to investigate the arena with the interaction mouse in it.

#### Carnosine-related genes in human phenotypes

To conduct the gene-based analyses, we obtained openly available summary statistics from GWAS performed in individuals of European descent (table S3). We then restricted the data, where possible, to SNPs with a minor allele frequency of 1%, with good imputation quality (imputation information score, >0.8), and that were represented in more than 70% of the total meta-analyzed sample size. We then calculated the eight genes of interest’s association with the chosen phenotypes using MAGMA (*39*). The gene-phenotype associations were calculated using the individual SNPs’ *P* values for association with the respective phenotypes. SNPs were assigned to a gene if their chromosomal location were within the start and end of the gene transcripts (i.e., the standard settings of MAGMA). We used the 1000 genomes CEU (Northern Europeans from Utah) population as the reference panel to correct for linkage disequilibrium.

#### Variant effect prediction

In total, Ensembl reported 23,292 SNPs in human GADL1, with 78% of them being intronic. Among the coding variants, 67% were presented as missense, 23% as synonymous, 5% as frameshift, 3% as stop gained, and 1% as in-frame deletion.

#### Structural analyses

For comparative protein structure analysis, the structures of mouse GADL1 (*25*) (PDB entry 6enz), human CSAD (PDB entry 2jis), and human GAD65 (*60*) (PDB entries 2okk) were used. To predict the binding mode of Glu into GAD65, its structure was re-refined and observed to contain the inhibitor chelidonic acid, based on which the Glu binding mode can easily be obtained because of the presence of two appropriately spaced carboxyl groups. Structures were superposed in COOT (*61*) and visualized in Chimera (*62*).

#### Hematoxylin and eosin staining

For the histochemistry studies, the organs were fixed with formalin. The samples were paraffin-embedded and sectioned into 40-mm-thick slices. For hematoxylin and eosin staining, sections were deparaffinized in Tissue-Clear II (2 × 10 min) and rehydrated using 100% ethanol (2 × 1 min each), followed by 96% ethanol (1 min) and then 70% ethanol for 1 min and double-distilled H_2_O (ddH_2_O) for 3 min. Sections were then dipped in 0.2% hematoxylin (Histolab) for 5 min, eosin for 1 min, followed by a 5-s wash with ddH_2_O. The sections were then rehydrated in 70% ethanol for 30 s, followed by dipping in 96% (2 min) and 100% ethanol (2 × 2 min). Last, the sections were dipped in xylene for 2 min, mounted on slides, and coverslipped. Hamamatsu slide scanner was used for scanning slides, and Aperio software was used to take pictures.

### Statistics

All data, unless specified otherwise, are presented as means ± SD. The growth curves (Fig. 1, B and C), enzyme activity assay (Fig. 1H), NMR (Fig. 3C), MRI, and animal behavior (Fig. 5, A to E) were analyzed with unpaired Student’s *t* test (two-tailed). Statistical significance was defined as *P* ≤ 0.05. Metabolomics data (Fig. 2 and fig. S4) were analyzed with unpaired Student’s *t* test (two-tailed) with Welch’s correction. Antioxidant levels (Fig. 4) were analyzed with a ratio, paired *t* test (two-tailed) per experimental setup.

### Study approval

The protocol used in this manuscript was approved by the Animal Studies Committee, Norwegian Food Safety Authority (Mattilsynet, permit number 9468).

## Supplementary Material

abb3713_Table_S2.xlsx

abb3713_SM.pdf

## SUPPLEMENTARY MATERIALS

Supplementary material for this article is available at http://advances.sciencemag.org/cgi/content/full/6/29/eabb3713/DC1

## Appendix

View/request a protocol for this paper from *Bio-protocol*.
