# Supplementary material for: GADL1 is a multifunctional decarboxylase with tissue-specific roles in β-alanine and carnosine production
Source: Sci Adv. 2020 Jul 17;6(29):eabb3713. doi: 10.1126/sciadv.abb3713 (PMC7367687; doi:10.1126/sciadv.abb3713)
Supplement: abb3713_SM.pdf [file abb3713_SM.pdf]

Science Advances
AAAS

advances.sciencemag.org/cgi/content/full/6/29/eabb3713/DC1

Supplementary Materials for

GADL1 is a multifunctional decarboxylase with tissue-specific roles in β-alanine and carnosine production

Elaheh Mahootchi, Selina Cannon Homaei, Rune Kleppe, Ingeborg Winge, Tor-Arne Hegvik, Roberto Megias-Perez, Christian Totland, Floriana Mogavero, Anne Baumann, Jeffrey Colm Glennon, Hrvoje Miletic, Petri Kursula, Jan Haavik*

*Corresponding author. Email: jan.haavik@uib.no

Published 17 July 2020, *Sci. Adv.* **6**, eabb3713 (2020)
DOI: 10.1126/sciadv.abb3713

The PDF file includes:

Tables S1, S3 to S5
Figs. S1 to S5
References

Other Supplementary Material for this manuscript includes the following:

(available at advances.sciencemag.org/cgi/content/full/6/29/eabb3713/DC1)

Table S2

Table S1 A. Top 25 upregulated genes in OB tissue samples from *Gadl1*$^{-/-}$ compared to *Gadl1*$^{+/+}$ mice.

ENSEMBL ID	Abbreviation	Name	Log2Fold	P-value
ENSMUSG00000032226	*Gcnt3*	Glucosaminyl (N-acetyl) transferase 3, mucin type	6.05	0.00084
ENSMUSG00000020159	*Gabrp*	γ-aminobutyric acid (GABA) A receptor, pi	5.77	0.01167
ENSMUSG00000085224	*Gm13425*	Predicted gene 13425	5.40	0.00262
ENSMUSG00000038805	*Six3*	Sine oculis-related homeobox 3	5.23	0.00077
ENSMUSG00000093894	*Ighv1-53*	Immunoglobulin heavy variable 1-53	5.22	0.02869
ENSMUSG00000096225	*Lhx8*	LIM homeobox protein 8	5.17	0.00055
ENSMUSG00000045620	*Odf3l1*	Outer dense fiber of sperm tails 3-like 1	4.97	0.00314
ENSMUSG00000061959	*Ces1e*	Carboxylesterase 1E	4.91	0.00405
ENSMUSG00000004341	*Gpx6*	Glutathione peroxidase 6	4.86	0.00372
ENSMUSG00000029866	*Kel*	Kell blood group	4.67	0.00028
ENSMUSG00000046975	*Olfr1020*	Olfactory receptor 1020	4.57	0.01397
ENSMUSG00000027902	*Chil6*	Chitinase like 6	4.55	0.00405
ENSMUSG00000024681	*Ms4a3*	Membrane-spanning 4-domains A3	4.49	0.02221
ENSMUSG00000112343	*Sfta3-ps*	Surfactant associated 3, pseudogene	4.38	0.02155
ENSMUSG00000082308	*Gm15770*	Predicted gene 15770	4.36	0.03261
ENSMUSG00000083986	*Gm12213*	Predicted gene 12213	4.36	0.02563
ENSMUSG00000090475	*Gm6245*	Predicted gene 6245	4.36	0.02527
ENSMUSG00000074665	*Bpifb4*	BPI fold containing family B4	4.35	0.01778
ENSMUSG00000073920	*Olfr661*	Olfactory receptor 661	4.30	0.02962
ENSMUSG00000058884	*Olfr1025-ps1*	Olfactory receptor 1025, pseudogene 1	4.30	0.03356
ENSMUSG00000087340	*Gm15228*	Predicted gene 15228	4.25	0.02579
ENSMUSG00000094872	*Igkv9-120*	Immunoglobulin kappa chain variable 9-120	4.22	0.01533
ENSMUSG00000066108	*Muc5b*	Mucin 5, subtype B, tracheobronchial	4.21	0.00077
ENSMUSG00000105906	*Iglc1*	Immunoglobulin lambda constant 1	4.18	0.01064
ENSMUSG00000095765	*Olfr741*	Olfactory receptor 741	4.16	0.00863

Table S1 B. Top 25 downregulated genes in OB tissue samples from *Gadl1*$^{-/-}$ compared to *Gadl1*$^{+/+}$ mice.

ENSEMBL ID	Abbreviation	Name	Log2Fold	P-value
ENSMUSG00000022485	*Hoxc5*	Homeobox Protein Hox C5	-8.25	0.00033
ENSMUSG00000038700	*Hoxb5*	Homeobox Protein Hox B5	-7.65	0.00325
ENSMUSG00000087658	*Hotairm1*	Hoxa transcript antisense RNA, myeloid-specific 1	-6.90	0.00079
ENSMUSG00000038253	*Hoxa5*	Homeobox Protein Hox A5	-6.46	0.00381
ENSMUSG00000056423	*Uts2b*	Urotensin-2B	-6.04	0.00188
ENSMUSG00000048763	*Hoxb3*	Homeobox Protein Hox B3	-5.99	0.00081
ENSMUSG00000001661	*Hoxc6*	Homeobox Protein Hox C6	-5.97	0.01110
ENSMUSG00000056468	*5730596B20Rik*	RIKEN cDNA 5730596B20 gene	-5.84	0.03447
ENSMUSG00000084844	*Hoxb3os*	Homeobox B3 and homeobox B2, opposite strand	-5.76	0.03083
ENSMUSG00000075394	*Hoxc4*	Homeobox Protein Hox C4	-5.67	0.00327
ENSMUSG00000085696	*Hoxaas3*	Hoxa cluster antisense RNA 3	-5.02	0.00906
ENSMUSG00000060738	*Prl7c1*	Prolactin 7c1	-4.85	0.00353
ENSMUSG00000005503	*Evx1*	Even-skipped homeobox 1	-4.65	0.03972
ENSMUSG00000067684	*Obp1a*	Odorant binding protein IA	-4.55	0.02233
ENSMUSG00000001670	*Tat*	Tyrosine aminotransferase	-4.48	0.02410
ENSMUSG00000103430	*Gm36996*	Predicted gene 36996	-4.45	0.01718
ENSMUSG00000108282	*Gm44317*	Predicted gene 44317	-4.41	0.01906
ENSMUSG00000026976	*Pax8*	Paired box 8	-4.29	0.00069
ENSMUSG00000038155	*Gstp2*	Glutathione S-transferase, pi 2	-4.28	0.03740
ENSMUSG00000042279	*H1foo*	H1.8 linker histone	-4.27	0.02103
ENSMUSG00000109753	*Gm45633*	Predicted gene 45633	-4.26	0.02596
ENSMUSG00000041333	*Mup4*	Major urinary protein 4	-4.26	0.02462
ENSMUSG00000073242	*Dnmt3aos*	DNA methyltransferase 3A, opposite strand	-4.18	0.02929
ENSMUSG00000074385	*Gm10684*	Predicted gene 10684	-4.14	0.00057
ENSMUSG00000029844	*Hoxa1*	Homeobox Protein Hox A1	-4.11	0.03148

Table S1 C. Transcript levels of 30 predicted genes close to the *Gadl1* locus (yellow: downregulated in knockout, green: upregulated in knockout, blank: not detected).

**Gene name**	**ENSEBML ID**	**Log2FoldChange**	**P-value**
Loc102633324 (Gm16142)	ENSMUSG00000087469	-1.145644169	0.331003828
Loc102633106 (Gm31014)			
Loc102633399 (Gm31234)			
Loc102633476 (Gm31292)			
Tgfbr2	ENSMUSG00000032440	0.153321074	0.11705412
Gm9385	ENSMUSG00000080848	-0.131387484	0.400319186
Gm5921	ENSMUSG00000074034	-0.062966245	0.955064616
Gm9385	ENSMUSG00000080848	-0.131387484	0.400319186
Rbms3	ENSMUSG00000039607	-0.041538879	0.816216697
Cmc1	ENSMUSG00000039163	0.043065942	0.678703046
Azi2	ENSMUSG00000039285	0.113416692	0.251718473
Stt3b	ENSMUSG00000032437	0.143339379	0.156764486
Gm18328			
Gm40582			
d73003k21rik			
Gm39447			
Gm40587			
Mir467h			
Gm9487			
4930428G15rik			
Gm30762			
Stmn1-rs1			
Gm31599			
Gm39449			
Gm18489			
Gm39452			
Loc102635502			
Gm36451			
Gm39450			
Loc108167721			
Gm4668			
Gm48038			
Gm31410			

Table S1 D. Transcript levels of ABAT or AGXT2 in the OB extracts of *Gadl1*$^{+/+}$ and *Gadl1*$^{-/-}$ mice. (yellow: downregulated in knockout, green: upregulated in knockout, blank: not detected).

**Gene name**	**ENSEMBL ID**	**Log2FoldChange**	**P-value**
Abat	ENSMUSG00000057880	0.063686739	0.554421048
Agxt2	ENSMUSG00000089678	-1.638874888	0.686109037

Table S3. Overview of the data used for genetic analysis using MAGMA software.

phenotype	Sample size*	Reference and data source
Neuropsychiatric		
ADHD**	53,293	Demontis et al. (64)
Alzheimer's disease	452,010	Jansen et al. (67)
ALS***	80,610	Nicolas et al.(70, 75)
Anorexia	72,517	Watson et al.(75)
Anxiety	18,186	Otowa et al. (71)
Autism	46,351	Grove et al. (66)
Bipolar disorder	51,710	Stahl et al. (72)
Depression	173,005	Wray et al. (76)
Educational attainment	766,345	Lee et al. (68)
Epilepsy	34,852	International League Against Epilepsy Consortium on Complex Epilepsies (79)
Parkinson's disease	482,730	Nalls et al. (69)
Schizophrenia	77,096	Ripke et al. (81)
SWB****	298,420	Okbay et al. (82)
Acetyl carnosine (in blood serum)	6279	Shin et al. (27)
AMD	33,976	Fritsche et al (65)
Body mass index	795,640	Yengo et al. (78)
Coronary heart disease	547,261	van der Harst & Verweij (74)
Muscular strength******	335,842	UKBIOBANK http://www.nealelab.is/uk-biobank http://ldsc.broadinstitute.org/
Type 2 diabetes	605,056	Xue et al. (77)
Kidney function*	567,460	Wuttke et al. (39)
UACR in diabetes**	5826	Teumer et al.(73)

*Maximum total sample size
** Attention-deficit/hyperactivity disorder
*** Amyotrophic lateral sclerosis
****Subjective well-being
***** Age-related macular degeneration
****** Right hand grip strength
* glomerular filtration rate estimated from serum creatinine (eGFR)
** urinary albumin-to-creatinine ratio among individuals with diabetes

Table S4. Analysis of mouse diet

Parameter analyzed	Result (g/kg)
1-Methylhistidine (Free)	<0.5
3-Methylhistidine (Free)	<0.5
Alanine (Free)	<0.5
Alpha-Aminoadipic acid (Free)	<0.5
Alpha-Amino-n-butyric acid (Free)	<0.5
Anserine (Free)	<0.5
Arginine (Free)	<0.5
Asparagine (Free)	0.535
Aspartic Acid (Free)	<0.5
β-Alanine (Free)	<0.5
β-Aminoisobutyric acid (Free)	<0.5
Carnosine	<0.5
Citrulline	<0.5
Cystathionine (Free)	<0.5
Cystin (Free)	<0.5
Delta-Hydroxylysine (Free)	<0.5
Ethanolamine	<0.5
γ-Amino-butyric acid (Free)	<0.5
Glutamic acid (Free)	<0.5
Glutamine (Free)	<0.5
Glycine (Free)	<0.5
Histidine (Free)	<0.5
Homocysteine (Free)	<0.5
Hydroxyproline (Free)	<0.5
Isoleucine (Free)	<0.5
Leucine (Free)	<0.5
Lysine (Free)	0.843
Methionine (Free)	<0.5
Ornithine (Free)	<0.5
Phenylalanine (Free)	<0.5
Phosphothanolamine (Free)	<0.5
Phosphoserine (Free)	<0.5
Proline (Free)	<0.5
Sarcosine (Free)	<0.5
Serine (Free)	<0.5
Taurine (Free)	<0.5
Threonine (Free)	<0.5
Tryptophan (Free)	<0.5
Tyrosine (Free)	<0.5
Urea	<0.5

Table S5. Sequences of PCR primers and sizes of PCR products.

Primer	Primer Sequence 5'-3'	PCR Product Size	
		**WT**	**KO**
136258Cre-HAA2	TCAGTTGAGAAGCCCTTCCTTGGTGTA	330 and 750 bp	166 bp
136249Cre-HAA2	CCTTGAACGTGGTTCTCTAGTAGCCACC		
136248Cre-HAA2	AGACCTGGTTAAGCAACTCTCCACTAACTCC		

Supplementary Figures

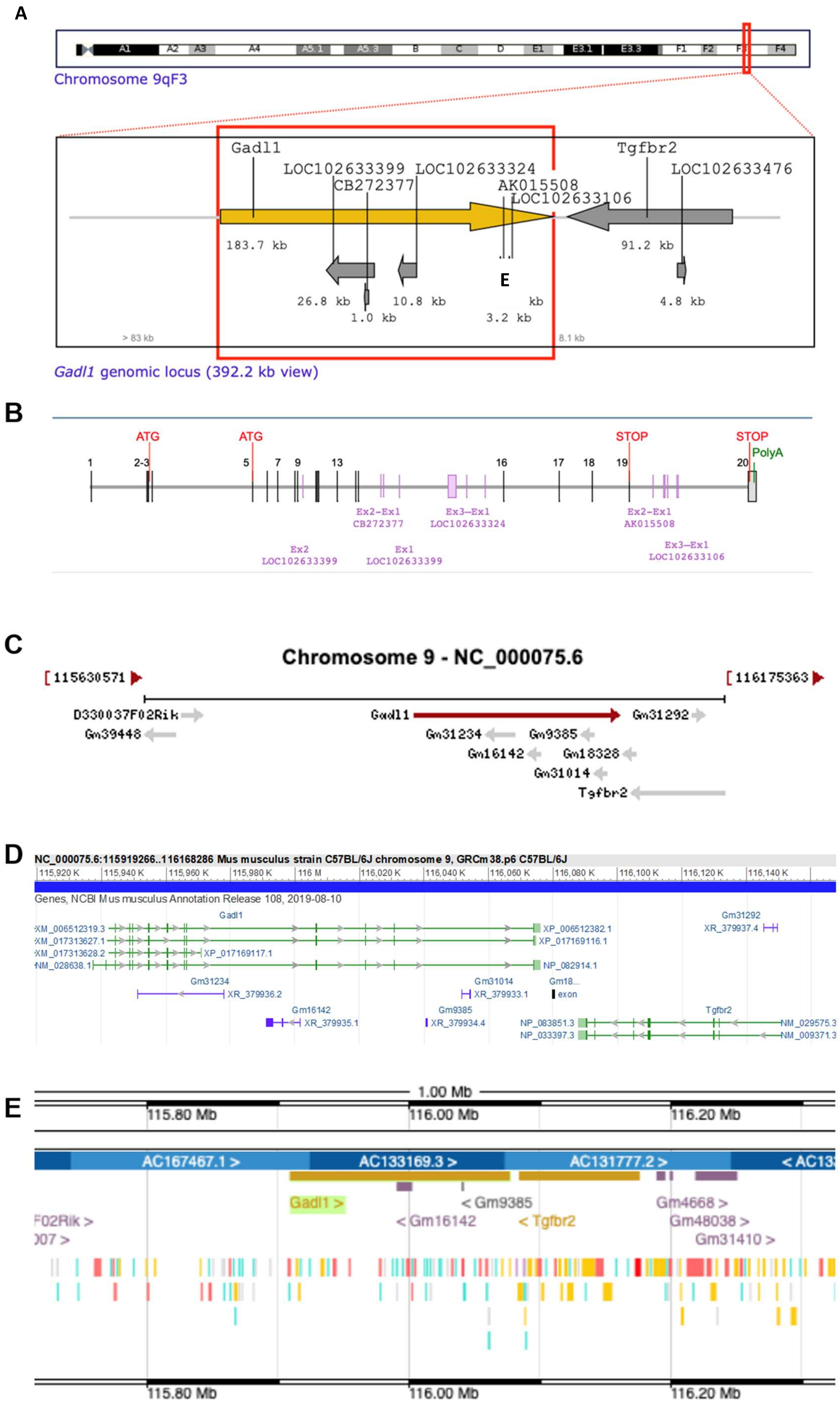

F

```
WT+/+    TTACTGATGAGAGTTCSCATCTTTGTGGATGGGGTTGTACTTAATGGACCTCAAACAGAT
KO-/-    GACCTGGT-AGAGTCCCATCTTTGTGGATGGGGTTGTACTTAATGGACCTCAAACAGAT
            *** * ***** * *******************************************

WT+/+    GTGAAAGCTGGAGAAAAATTTGTTGAAGAAGCCTGTAGGCTCATAATGGAAGAGGTGGTT
KO-/-    GTGAAAGCTGGAGAAAAATTTGTTGAAGAAGCCTGTAGGCTCATAATGGAAGAGGTGGTT
         ************************************************************

WT+/+    TTGAAAGCTACGGACGTCAATGAAAAGGTATGTGAGTGGCAGCCCCCTGAGCAGCTGAGG
KO-/-    TTGAAAGCTACGGACGTCAATGAAAAGGTATGTGAGTGGCAGCCCCCTGAGCAGCTGAGG
         ************************************************************

WT+/+    CAGCTCCTTGACCTGGAGATGAGGGACACAGGCGAGTCACAGGACAAGCTGCTGAAACTC
KO-/-    CAGCTCCTTGACCTGGAGATGAGGGACACAGGCGAGTCACAGGACAAGCTGCTGAAACTC
         ************************************************************

WT+/+    TGCCAAGACGTCATCCACTTCAGCGTCAAAACCAACCATCCAAGATTTTTCAACCAGCTC
KO-/-    TGCCAAGACGTCATCCACTTCAGCGTCAAAACCA--------------------------
         **********************************

WT+/+    TATGCTGGCCTTGATTACTACTCCTTGGCGGCCCGGATTATAACAGAGGCGCTGAATCCA
KO-/-    ------------------------------------------------------------

WT+/+    AGCATTTATACGTATGAGGTGTCCCCAGTGTTTCTGTTAGTGGAAGAGGCGGTTCTGAAG
KO-/-    ------------------------------------------------------------

WT+/+    AAAATGATTGAATGTGTTGGCTGGAAAGAAGGGGATGGAATATTTAACCCAGGTGGCTCA
KO-/-    ----------------------------------------------------GTGGCTCA
                                                             ********

WT+/+    GTGTCCAATATGTGTGCAATGAATTTAGCTAGATACAGACATTGCCCTGATATCAAAGAG
KO-/-    GTGTCCAATATGTGTGCAATGAATTTAGCTAGATACAGACATTGCCCTGATATCAAAGAG
         ************************************************************

WT+/+    AAAGGCCTGTCTGGTTTGCCAAGATTAATCCTTTTCACATCGGCAGAGTGCCATTACTCC
KO-/-    AAAGGCCTGTCTGGTTTGCCAAGATTAATCCTTTTCACATCGGCAGAGTGCCATTACTCC
         ************************************************************

WT+/+    TGAAAGAAAGCAAA
KO-/-    TGAAAGAAAGCA--
         ************
```

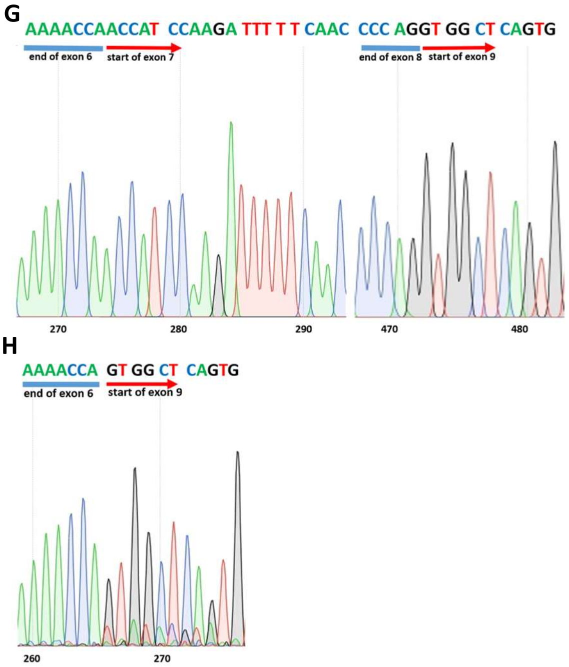

Fig. S1. Overview of the mouse *Gadl1* locus (A, B, C, D and E) and sequencing alignment of *Gadl1*$^{+/+}$ and *Gadl1*$^{-/-}$mRNA confirming that exon 7 and 8 are missing from the *Gadl1*$^{-/-}$ muscle transcript (F, G and H). **(A)** *Gadl1* genomic locus in mouse chromosome as analyzed at Genoway, **(B)** overview of exon organization using Genoway software, **(C)** overview of *Gadl1* locus from NCBI gene database, **(D)** *Gadl1* and closest neighboring genes (NCBI), **(E)** *Gadl1* and closest neighboring genes (EMBL-EBI). **(F)** Alignment of mRNA sequences of the *Gadl1*$^{+/+}$ and *Gadl1*$^{-/-}$ mice. Cluster omega was used for alignment (https://www.ebi.ac.uk/Tools/msa/muscle/). **(G-H)** Sequencing chromatogram of **(G)** *Gadl1*$^{+/+}$ and **(H)** *Gadl1*$^{-/-}$ mice. Exon 7 and 8 are missing in the *Gadl1*$^{-/-}$ mice mRNA. Created in SnapGene (v4.2, GSL Biotech LLC, San Diego, CA 92108, USA).

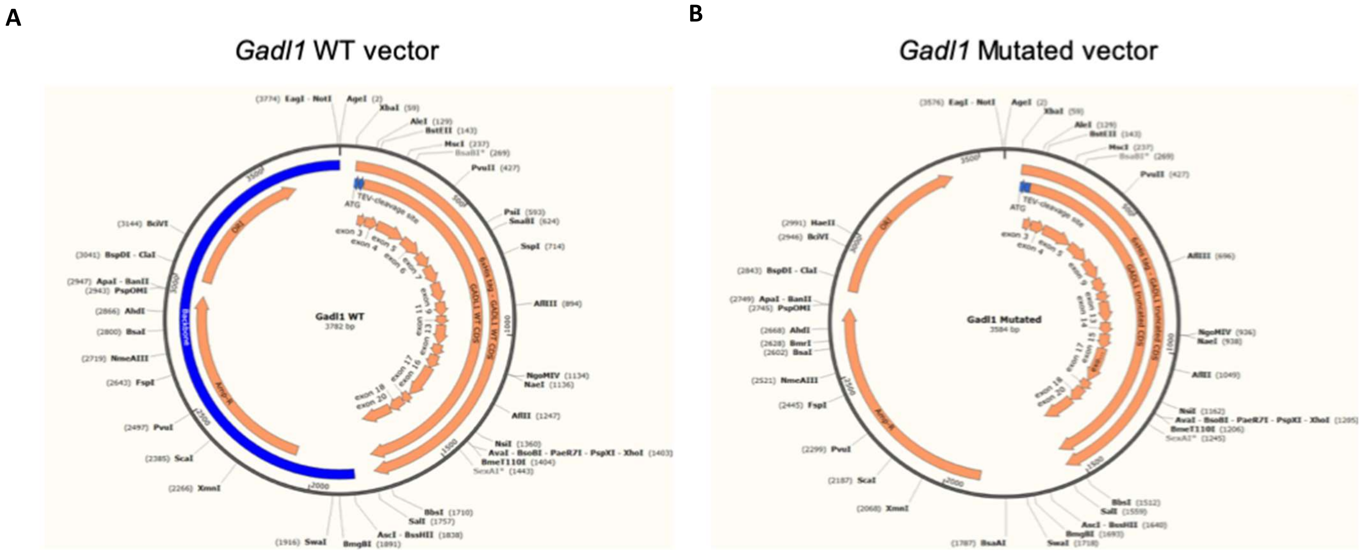

Fig. S2. Vectors designed for *Gadl1* wild type (WT) (A) and mutated (B). The vectors were made in BL21 E-coli. The proteins were expressed and purified using Histidine-tag. Created using SnapGene (v4.2, GSL Biotech).

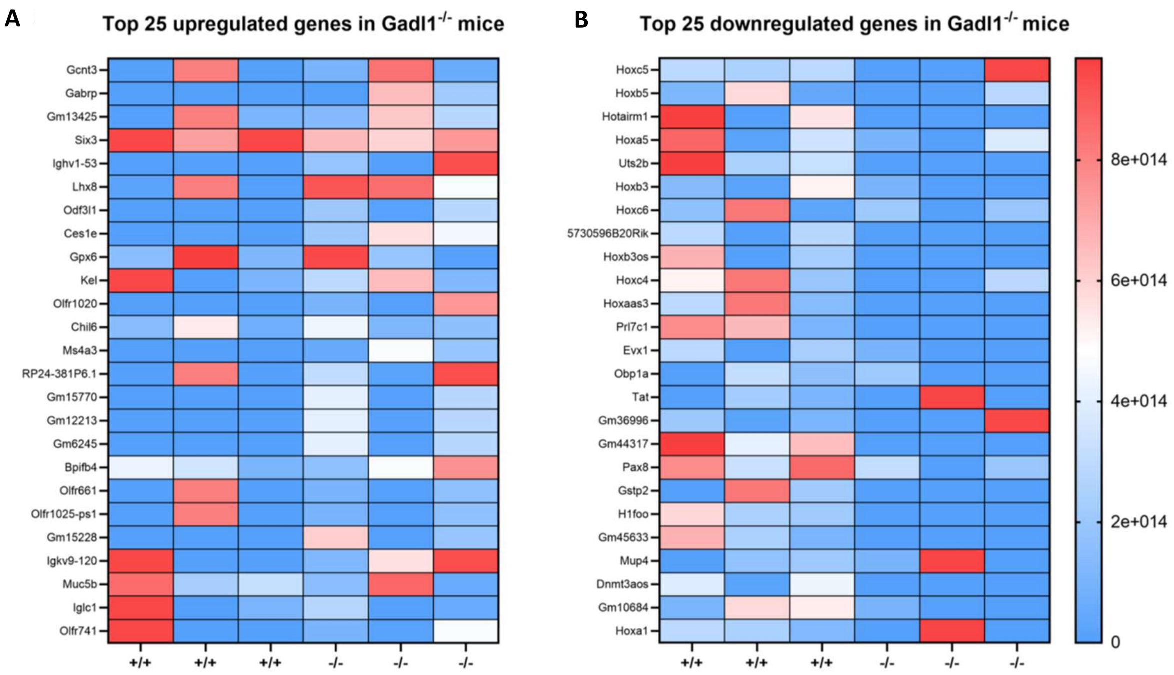

Fig. S3. Top 25 up (A) and down (B) regulated genes in olfactory bulb when comparing *Gadl1*$^{-/-}$ (n =3) to *Gadl1*$^{+/+}$ (n = 3).

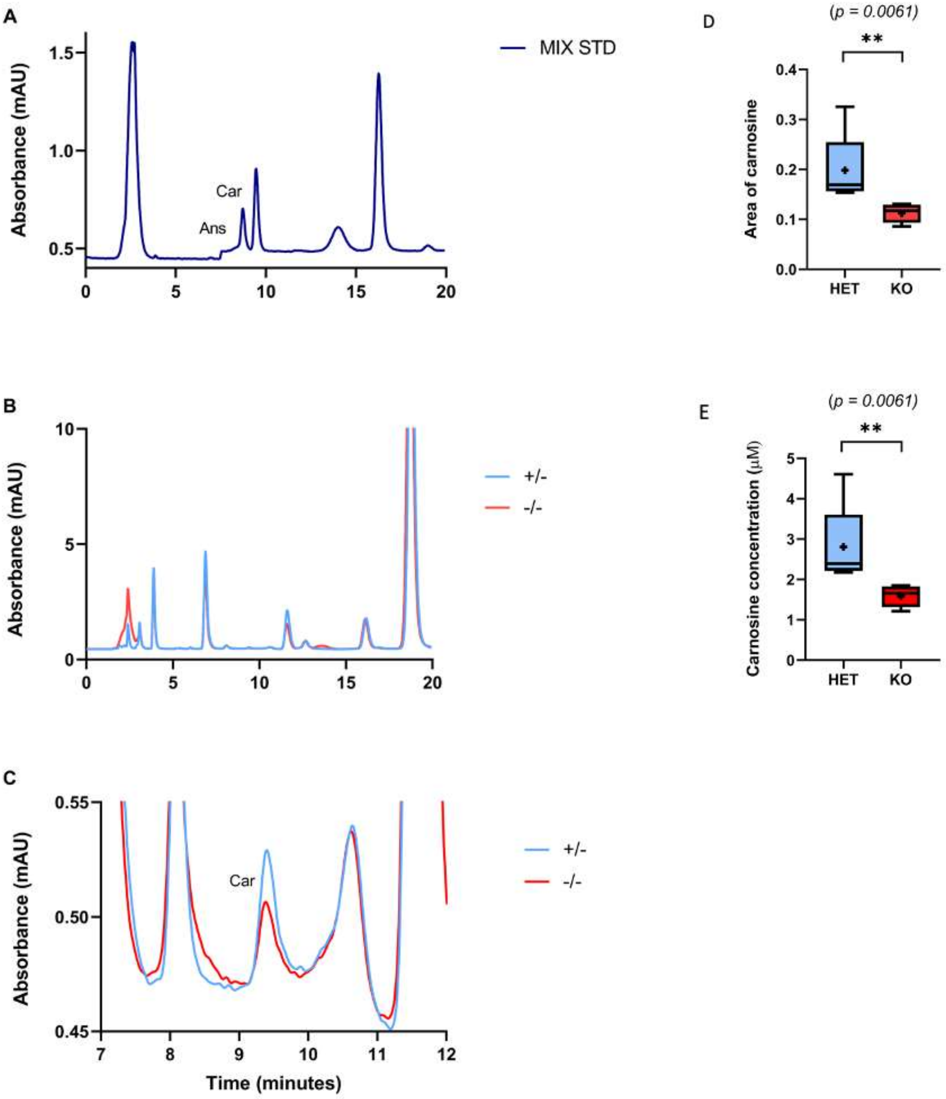
A
MIX STD
Absorbance (mAU)
1.5
1.0
0.5
Car
Ans
0
5
10
15
20
D
(p = 0.0061)
**
Area of carnosine
0.4
0.3
0.2
0.1
0.0
HET
KO
B
+/-
-/-
Absorbance (mAU)
10
5
0
0
5
10
15
20
E
(p = 0.0061)
**
Carnosine concentration (μM)
5
4
3
2
1
0
HET
KO
C
+/-
-/-
Car
Absorbance (mAU)
0.55
0.50
0.45
7
8
9
10
11
12
Time (minutes)

F

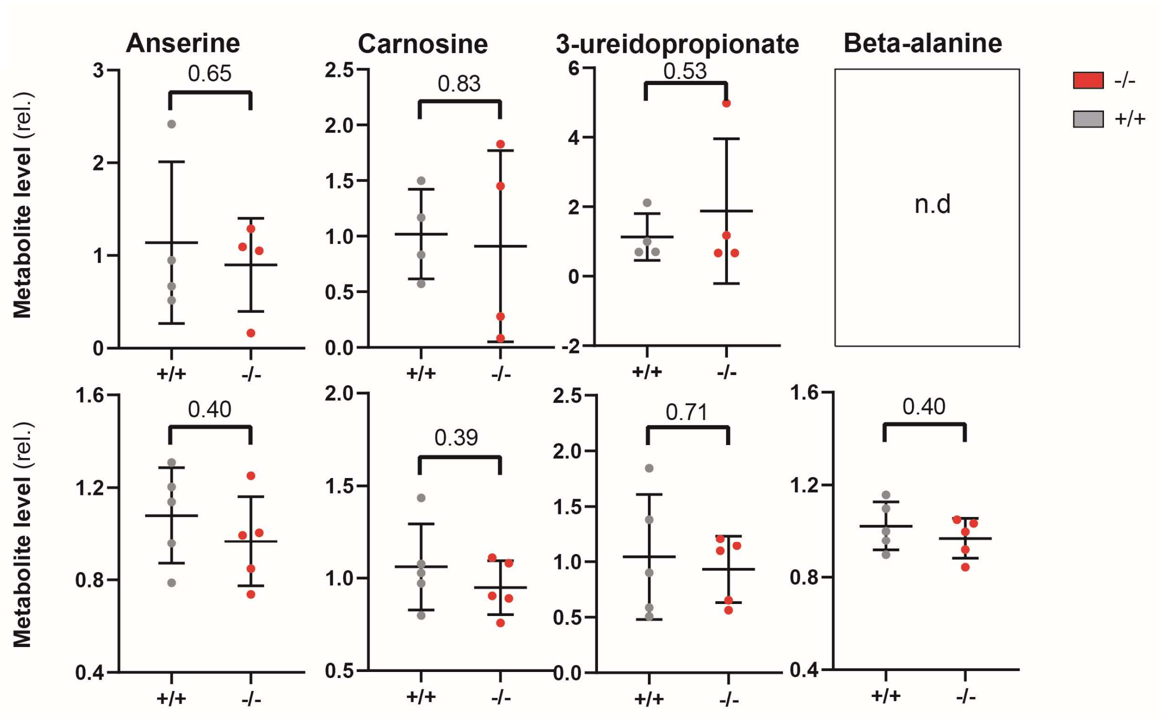
Anserine
Carnosine
3-ureidopropionate
Beta-alanine
-/-
+/+
Serum
Blood
Metabolite level (rel.)
0.65
0.83
0.53
n.d
0.40
0.39
0.71
0.40
+/+
-/-

G

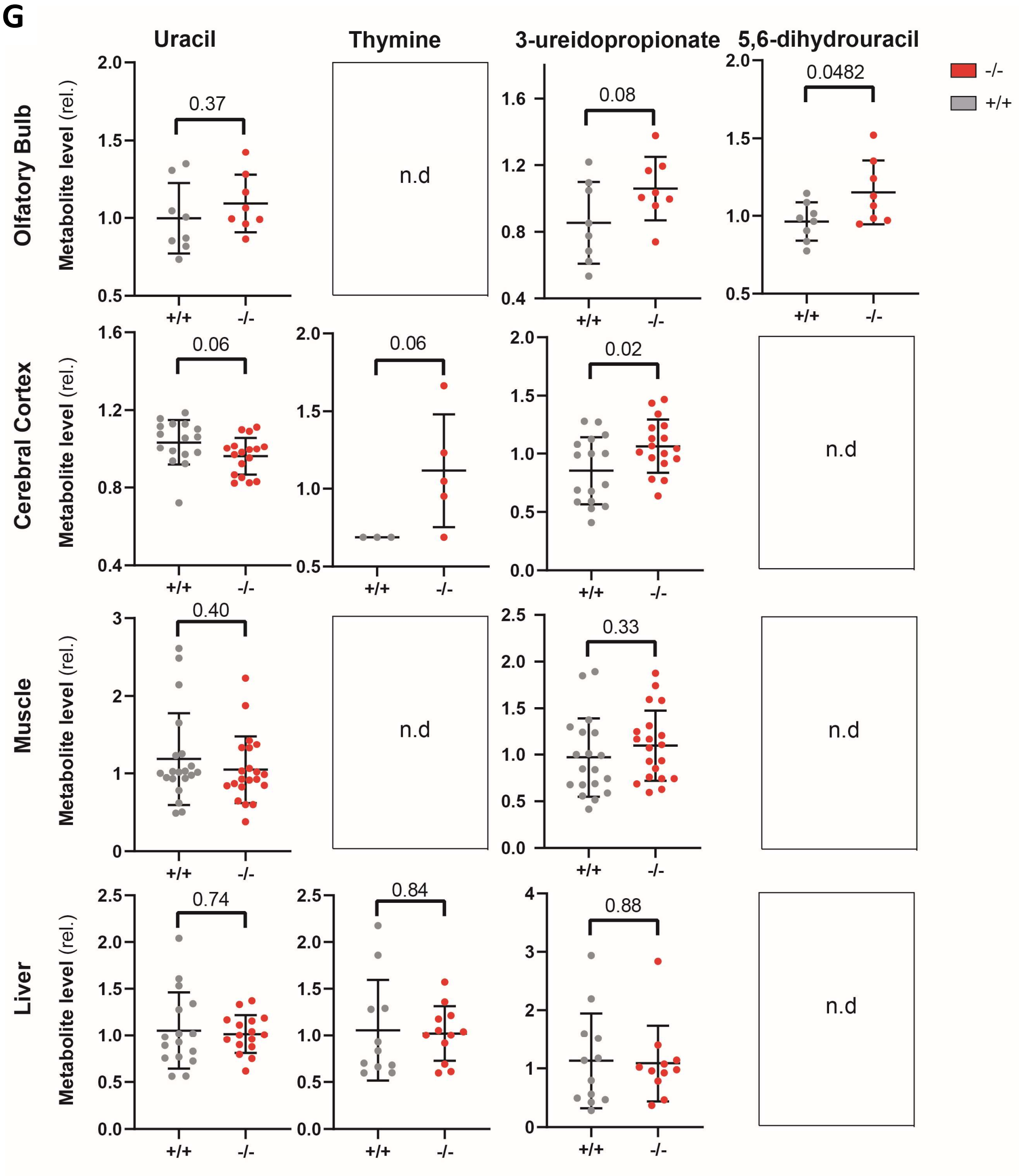
Uracil
Thymine
3-ureidopropionate
5,6-dihydrouracil
-/-
+/+
Olfatory Bulb
Cerebral Cortex
Muscle
Liver
Metabolite level (rel.)
0.37
n.d
0.08
0.0482
0.06
0.06
0.02
n.d
0.40
n.d
0.33
n.d
0.74
0.84
0.88
n.d
+/+
-/-

H

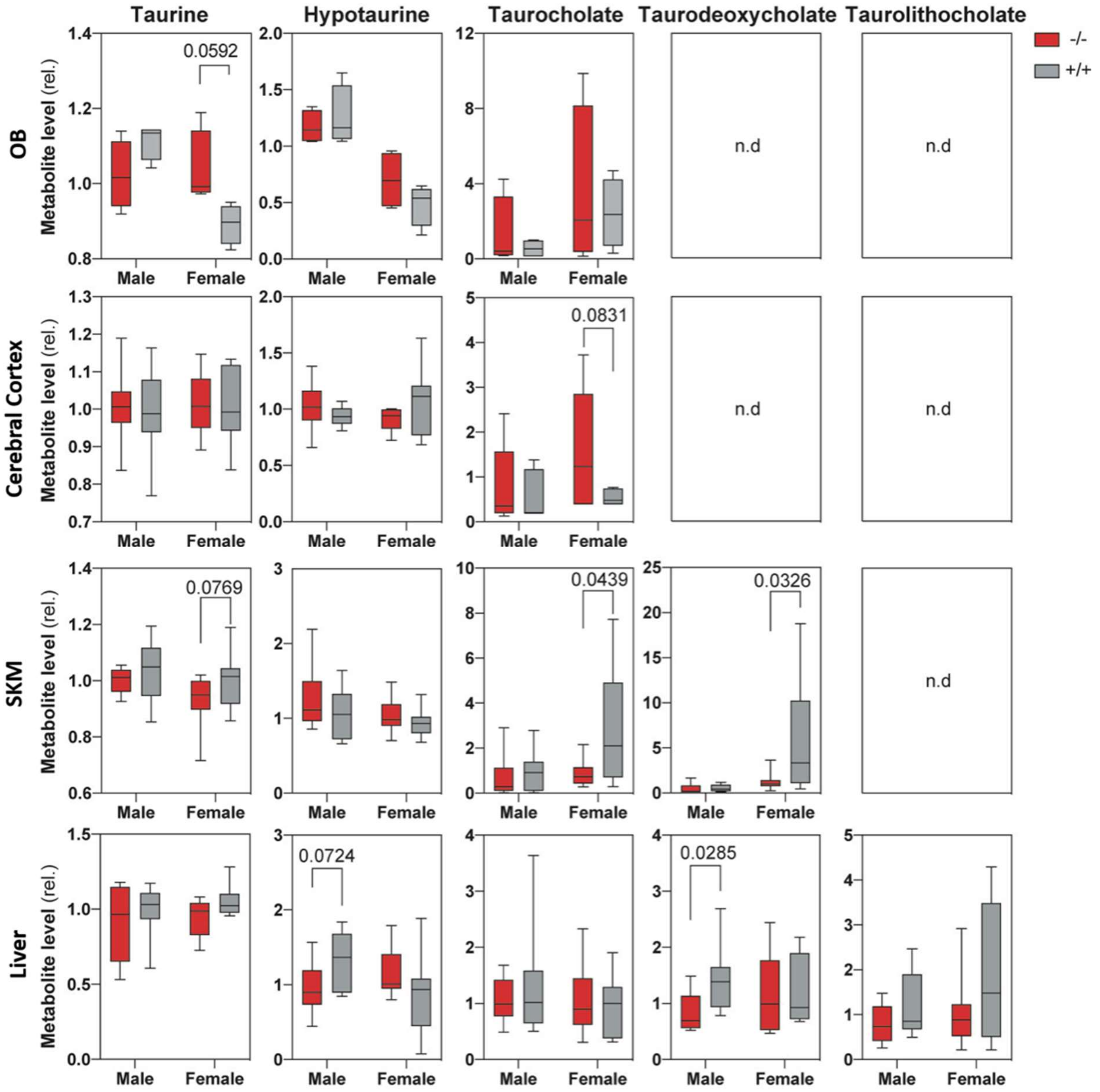

Taurine
Hypotaurine
Taurocholate
Taurodeoxycholate
Taurolithocholate
-/-
+/+
OB
Cerebral Cortex
SKM
Liver
Metabolite level (rel.)
Male
Female
n.d
0.0592
0.0831
0.0769
0.0439
0.0326
0.0724
0.0285

I

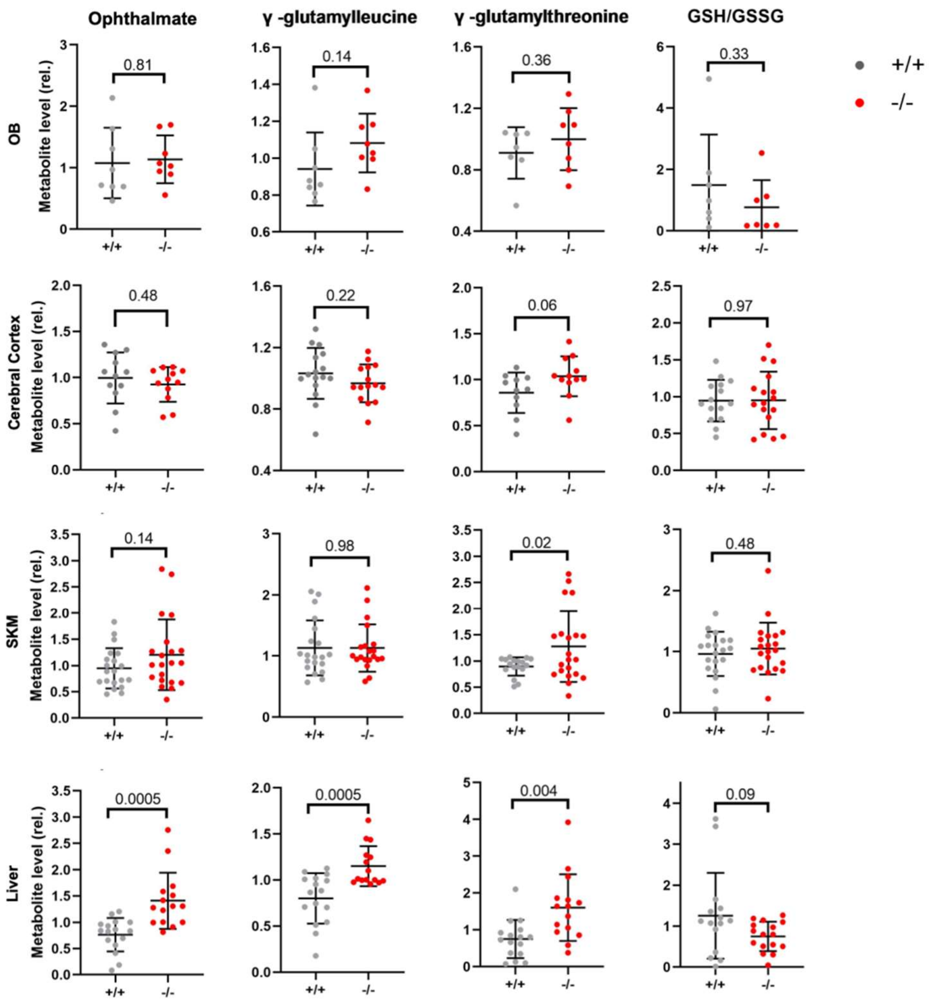
Ophthalmate
γ -glutamylleucine
γ -glutamylthreonine
GSH/GSSG
+/+
-/-
OB
Cerebral Cortex
SKM
Liver
Metabolite level (rel.)
0.81
0.14
0.36
0.33
0.48
0.22
0.06
0.97
0.14
0.98
0.02
0.48
0.0005
0.0005
0.004
0.09

J

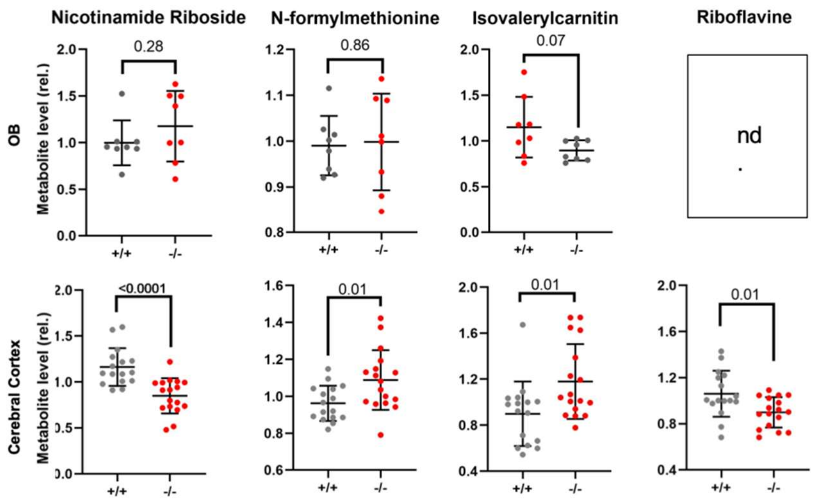
Nicotinamide Riboside
N-formylmethionine
Isovalerylcarnitin
Riboflavine
OB
Cerebral Cortex
Metabolite level (rel.)
0.28
0.86
0.07
nd
<0.0001
0.01
0.01
0.01
+/+
-/-

K

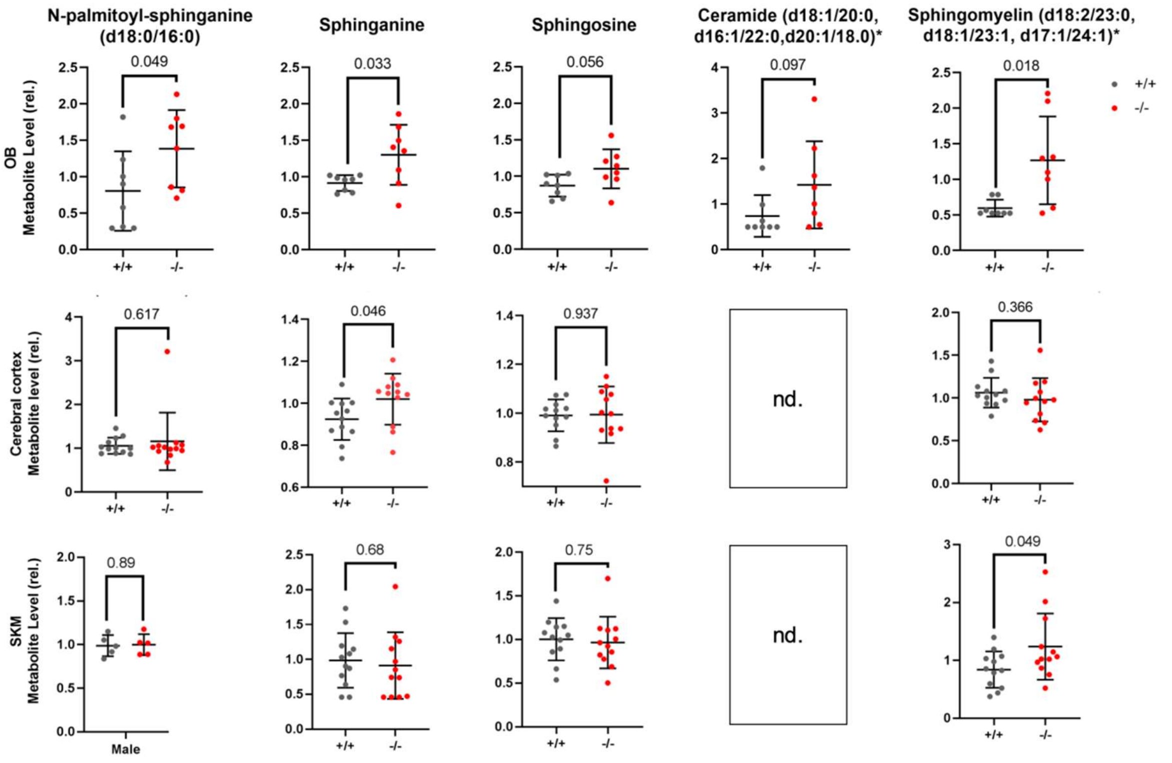

Fig. S4. Metabolite levels in mouse tissues. (A, B, C, D and E) Measurements of carnosine peptides in extracts of cerebral cortex from female *Gadl1*$^{+/-}$ (n=5) and *Gadl1*$^{-/-}$ (n=5) mice (18-22 weeks) using an isocratic RP-HPLC system. Injection of 20µl **(A)** L-anserine nitrate salt and L-carnosine, and **(B and C)** cerebral cortex lysates from female *Gadl1*$^{+/-}$ (n=5) and *Gadl1*$^{-/-}$ (n=5) mice (18-22 weeks). The box plots show **(D)** mean area and **(E)** relative level of carnosine concentration in cortex extracts. The decrease in carnosine levels was 40.46 ± 12.18 % from *Gadl1*$^{+/-}$ to *Gadl1*$^{-/-}$. Median is presented as a line and mean as a plus (+) sign. Whiskers are drawn from min to max value. Statistical significance (p<0.05) was determined with a ratio, paired t-test and significant differences are indicated with an asterisk (*). Relative levels of **(F)** 3-ureido-propionate, β-alanine, anserine, and carnosine in whole blood and serum (n=5). Relative levels of various metabolites in OB, cerebral cortex, SKM, and liver tissue extracts from *Gadl1*$^{+/+}$ (grey) and *Gadl1*$^{-/-}$ (red) mice (n=5-21); **(G)** uracil and thymine derivatives, **(H)** taurine and taurine derivatives, **(I)** Glutamate peptides and related oxidative stress markers, **(J)** Nicotinamide riboside, N-formylmethionine, isovalerylcarnitine, riboflavin, **(K)** sphingolipids and ceramide.

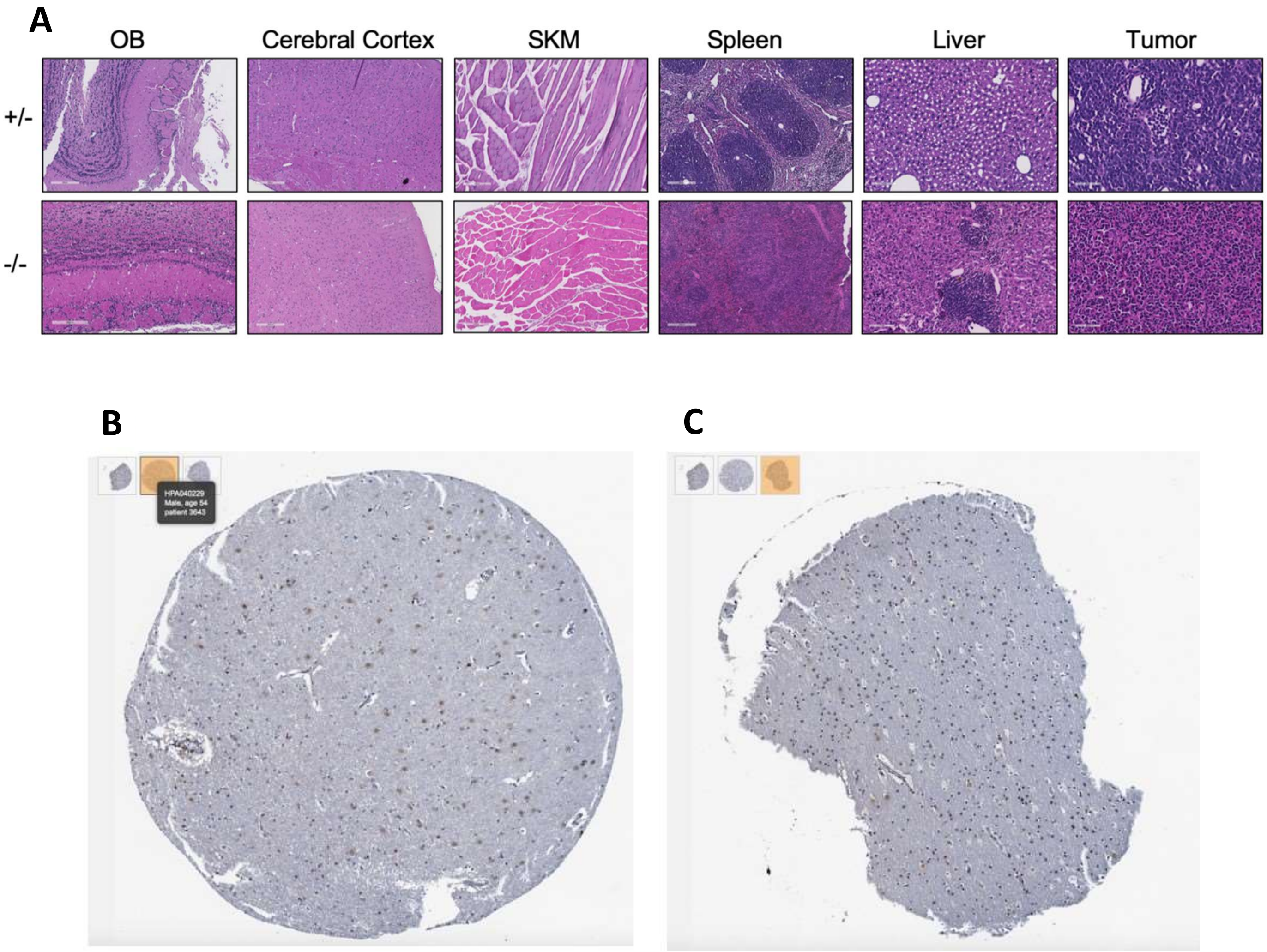

Fig. S5. Tissue distribution of GADL1 (A) Hematoxylin and Eosin (HE) staining of mouse *Gadl1* $^{+/-}$ and *Gadl1*$^{-/-}$ OB, cerebral cortex, SKM, spleen, liver and tumor. (B-C) Immunohistochemistry for GADL1 of (B) grey and (C) white matter using commercial antibody adapted from Human Protein Atlas.

Links to databases:
Human Protein Atlas
https://www.proteinatlas.org/ENSG00000144644-GADL1/tissue

alliance of genome resources
http://www.informatics.jax.org/marker/MGI:1920998

REFERENCES AND NOTES

1. A. A. Boldyrev, G. Aldini, W. Derave, Physiology and pathophysiology of carnosine. *Physiol. Rev.* **93**, 1803–1845 (2013).
2. R. Chaleckis, I. Murakami, J. Takada, H. Kondoh, M. Yanagida, Individual variability in human blood metabolites identifies age-related differences. *Proc. Natl. Acad. Sci. U.S.A.* **113**, 4252–4259 (2016).
3. A. R. Hipkiss, Is carnosine a naturally occurring suppressor of oxidative damage in olfactory neurones? *Rejuvenation Res.* **7**, 253–255 (2004).
4. T. L. Perry, S. Hansen, B. Tischler, R. Bunting, K. Berry, Carnosinemia — A new metabolic disorder associated with neurologic disease and mental defect. *N. Engl. J. Med.* **277**, 1219–1227 (1967).
5. K. Wisniewski, L. Fleisher, D. Rassin, H. Lassmann, Neurological disease in a child with carnosinase deficiency. *Neuropediatrics* **12**, 143–151 (1981).
6. J. H. Cararo, E. L. Streck, P. F. Schuck, G. da C Ferreira, Carnosine and related peptides: Therapeutic potential in age-related disorders. *Aging Dis.* **6**, 369–379 (2015).
7. R. M. Hobson, B. Saunders, G. Ball, R. C. Harris, C. Sale, Effects of β-alanine supplementation on exercise performance: A meta-analysis. *Amino Acids* **43**, 25–37 (2012).
8. A. E. Smith, A. A. Walter, J. L. Graef, K. L. Kendall, J. R. Moon, C. M. Lockwood, D. H. Fukuda, T. W. Beck, J. T. Cramer, J. R. Stout, Effects of β-alanine supplementation and high-intensity interval training on endurance performance and body composition in men; a double-blind trial. *J. Int. Soc. Sports Nutr.* **6**, 5 (2009).
9. B. Saunders, K. Elliott-Sale, G. G. Artioli, P. A. Swinton, E. Dolan, H. Roschel, C. Sale, B. Gualano, β-alanine supplementation to improve exercise capacity and performance: A systematic review and meta-analysis. *Br. J. Sports Med.* **51**, 658–669 (2016).
10. M. G. Chez, C. P. Buchanan, M. C. Aimonovitch, M. Becker, K. Schaefer, C. Black, J. Komen, Double-blind, placebo-controlled study of L-carnosine supplementation in children with autistic spectrum disorders. *J. Child Neurol.* **17**, 833–837 (2002).
11. T. Murakami, M. Furuse, The impact of taurine- and beta-alanine-supplemented diets on behavioral and neurochemical parameters in mice: Antidepressant versus anxiolytic-like effects. *Amino Acids* **39**, 427–434 (2010).

12. M. Schön, A. Mousa, M. Berk, W. L. Chia, J. Ukropec, A. Majid, B. Ukropcová, B. de Courten, The potential of carnosine in brain-related disorders: A comprehensive review of current evidence. *Nutrients* **11**, 1196 (2019).
13. G. G. Artioli, B. Gualano, A. Smith, J. Stout, A. H. Lanch Jr., Role of β-alanine supplementation on muscle carnosine and exercise performance. *Med. Sci. Sports Exerc.* **42**, 1162–1173 (2010).
14. E. J. Anderson, G. Vistoli, L. A. Katunga, K. Funai, L. Regazzoni, T. Blake Monroe, E. Gilardoni, L. Cannizzaro, M. Colzani, D. De Maddis, G. Rossoni, R. Canevotti, S. Gagliardi, M. Carini, G. Aldini, A carnosine analog mitigates metabolic disorders of obesity by reducing carbonyl stress. *J. Clin. Invest.* **128**, 5280–5293 (2018).
15. M. Dunnett, R. C. Harris, Influence of oral β-alanine and L-histidine supplementation on the carnosine content of the *gluteus medius*. *Equine Vet. J. Suppl.* **30**, 499–504 (1999).
16. E. P. Rhee, J. E. Ho, M.-H. Chen, D. Shen, S. Cheng, M. G. Larson, A. Ghorbani, X. Shi, I. T. Helenius, C. J. O'Donnell, A. L. Souza, A. Deik, K. A. Pierce, K. Bullock, G. A. Walford, R. S. Vasan, J. C. Florez, C. Clish, J.-R. J. Yeh, T. J. Wang, R. E. Gerszten, A genome-wide association study of the human metabolome in a community-based cohort. *Cell Metab.* **18**, 130–143 (2013).
17. R. Zrenner, H. Riegler, C. R. Marquard, P. R. Lange, C. Geserick, C. E. Bartosz, C. T. Chen, R. D. Slocum, A functional analysis of the pyrimidine catabolic pathway in Arabidopsis. *New Phytol.* **183**, 117–132 (2009).
18. F. Schmitzberger, M. L. Kilkenny, C. M. C. Lobley, M. E. Webb, M. Vinkovic, D. Matak-Vinkovic, M. Witty, D. Y. Chirgadze, A. G. Smith, C. Abell, T. L. Blundell, Structural constraints on protein self-processing in L-aspartate-α-decarboxylase. *EMBO J.* **22**, 6193–204 (2003).
19. S. Chopra, H. Pai, A. Ranganathan, Expression, purification, and biochemical characterization of *Mycobacterium tuberculosis* aspartate decarboxylase, PanD. *Protein Expr. Purif.* **25**, 533–540 (2002).
20. S. Suidasari, J. Stautemas, S. Uragami, N. Yanaka, W. Derave, N. Kato, Carnosine content in skeletal muscle is dependent on vitamin B6 status in rats. *Front. Nutr.* **2**, 39 (2015).
21. C.-H. Chen, C.-S. Lee, M.-T. M. Lee, W.-C. Ouyang, C.-C. Chen, M.-Y. Chong, J.-Y. Wu, H. K.-L. Tan, Y.-C. Lee, L.-J. Chuo, N.-Y. Chiu, H.-Y. Tsang, T.-J. Chang, F.-W. Lung, C.-

H. Chiu, C.-H. Chang, Y.-S. Chen, Y.-M. Hou, C.-C. Chen, T.-J. Lai, C.-L. Tung, C.-Y. Chen, H.-Y. Lane, T.-P. Su, J. Feng, J.-J. Lin, C.-J. Chang, P.-R. Teng, C.-Y. Liu, C.-K. Chen, I.-C. Liu, J.-J. Chen, T. Lu, C.-C. Fan, C.-K. Wu, C.-F. Li, K. H.-T. Wang, L. S.-H. Wu, H.-L. Peng, C.-P. Chang, L.-S. Lu, Y.-T. Chen, A. T.-A. Cheng; Taiwan Bipolar Consortium, Variant GADL1 and response to lithium therapy in bipolar I disorder. *N. Engl. J. Med.* **370**, 119–128 (2014).

22. C. Cruceanu, M. Alda, P. A. Dion, G. Turecki, G. A. Rouleau, No evidence for *GADL1* variation as a bipolar disorder susceptibility factor in a Caucasian lithium-responsive cohort. *Am. J. Psychiatry* **172**, 94–95 (2015).
23. P. Liu, X. Ge, H. Ding, H. Jiang, B. M. Christensen, J. Li, Role of glutamate decarboxylase-like protein 1 (GADL1) in taurine biosynthesis. *J. Biol. Chem.* **287**, 40898–40906 (2012).
24. I. Winge, K. Teigen, A. Fossbakk, E. Mahootchi, R. Kleppe, F. Skoldberg, O. Kämpe, J. Haavik, Mammalian CSAD and GADL1 have distinct biochemical properties and patterns of brain expression. *Neurochem. Int.* **90**, 173–184 (2015).
25. A. Raasakka, E. Mahootchi, I. Winge, W. Luan, P. Kursula, J. Haavik, Structure of the mouse acidic amino acid decarboxylase GADL1. *Acta Crystallogr. F. Struct. Biol. Commun.* **74** (Pt 1), 65–73 (2018).
26. S.-Y. Shin, E. B. Fauman, A.-K. Petersen, J. Krumsiek, R. Santos, J. Huang, M. Arnold, I. Erte, V. Forgetta, T.-P. Yang, K. Walter, C. Menni, L. Chen, L. Vasquez, A. M. Valdes, C. L. Hyde, V. Wang, D. Ziemek, P. Roberts, L. Xi, E. Grundberg; The Multiple Tissue Human Expression Resource (Mu THER) Consortium, M. Waldenberger, J. B. Richards, R. P. Mohney, M. V. Milburn, S. L. John, J. Trimmer, F. J. Theis, J. P. Overington, K. Suhre, M. J. Brosnan, C. Gieger, G. Kastenmüller, T. D. Spector, N. Soranzo, An atlas of genetic influences on human blood metabolites. *Nat. Genet.* **46**, 543–550 (2014).
27. M. Kanehisa, S. Goto, KEGG: Kyoto encyclopedia of genes and genomes. *Nucleic Acids Res.* **28**, 27–30 (2000).
28. The Gene Ontology Consortium, Gene Ontology Consortium: Going forward. *Nucleic Acids Res.* **43** (Database issue), D1049–D1056 (2015).
29. R. M. Kream, F. L. Margolis, Olfactory marker protein: Turnover and transport in normal and regenerating neurons. *J. Neurosci.* **4**, 868–879 (1984).

30. T.-N. Wu, C.-K. Chen, C.-S. Lee, B.-J. Wu, H.-J. Sun, C.-H. Chang, C.-Y. Chen, L. S.-H. Wu, A. T.-A. Cheng, Lithium and GADL1 regulate glycogen synthase kinase-3 activity to modulate *KCTD12* expression. *Sci. Rep.* **9**, 10255 (2019).
31. R. Percudani, A. Peracchi, A genomic overview of pyridoxal-phosphate-dependent enzymes. *EMBO Rep.* **4**, 850–854 (2003).
32. G. Fenalti, R. H. P. Law, A. M. Buckle, C. Langendorf, K. Tuck, C. J. Rosado, N. G. Faux, K. Mahmood, C. S. Hampe, J. P. Banga, M. Wilce, J. Schmidberger, J. Rossjohn, O. El-Kabbani, R. N. Pike, A. I. Smith, I. R. Mackay, M. J. Rowley, J. C. Whisstock, GABA production by glutamic acid decarboxylase is regulated by a dynamic catalytic loop. *Nat. Struct. Mol. Biol.* **14**, 280–286 (2007).
33. E. Park, S. Y. Park, C. Dobkin, G. Schuller-Levis, Development of a novel cysteine sulfinic acid decarboxylase knockout mouse: Dietary taurine reduces neonatal mortality. *J. Amino Acids* **2014**, 346809 (2014).
34. J. E. Dominy Jr., C. R. Simmons, L. L. Hirschberger, J. Hwang, R. M. Coloso, M. H. Stipanuk, Discovery and characterization of a second mammalian thiol dioxygenase, cysteamine dioxygenase. *J. Biol. Chem.* **282**, 25189–25198 (2007).
35. A. A. Boldyrev, Carnosine: New concept for the function of an old molecule. *Biochemistry (Mosc.)* **77**, 313–326 (2012).
36. M. N. Nikolova-Karakashian, M. B. Reid, Sphingolipid metabolism, oxidant signaling, and contractile function of skeletal muscle. *Antioxid. Redox Signal.* **15**, 2501–2517 (2011).
37. T. D. Wade, S. Gordon, S. Medland, C. M. Bulik, A. C. Heath, G. W. Montgomery, N. G. Martin, Genetic variants associated with disordered eating. *Int. J. Eat. Disord.* **46**, 594–608 (2013).
38. M. Wuttke, Y. Li, M. Li, K. B. Sieber, M. F. Feitosa, M. Gorski, A. Tin, L. Wang, A. Y. Chu, A. Hoppmann, H. Kirsten, A. Giri, J.-F. Chai, G. Sveinbjornsson, B. O. Tayo, T. Nutile, C. Fuchsberger, J. Marten, M. Cocca, S. Ghasemi, Y. Xu, K. Horn, D. Noce, P. J. van der Most, S. Sedaghat, Z. Yu, M. Akiyama, S. Afaq, T. S. Ahluwalia, P. Almgren, N. Amin, J. Ärnlöv, S. J. L. Bakker, N. Bansal, D. Baptista, S. Bergmann, M. L. Biggs, G. Biino, M. Boehnke, E. Boerwinkle, M. Boissel, E. P. Bottinger, T. S. Boutin, H. Brenner, M. Brumat, R. Burkhardt, A. S. Butterworth, E. Campana, A. Campbell, H. Campbell, M. Canouil, R. J. Carroll, E. Catamo, J. C. Chambers, M.-L. Chee, M.-L. Chee, X. Chen, C.-Y. Cheng, Y.

Cheng, K. Christensen, R. Cifkova, M. Ciullo, M. P. Concas, J. P. Cook, J. Coresh, T. Corre, C. F. Sala, D. Cusi, J. Danesh, E. W. Daw, M. H. de Borst, A. De Grandi, R. de Mutsert, A. P. J. de Vries, F. Degenhardt, G. Delgado, A. Demirkan, E. D. Angelantonio, K. Dittrich, J. Divers, R. Dorajoo, K.-U. Eckardt, G. Ehret, P. Elliott, K. Endlich, M. K. Evans, J. F. Felix, V. H. X. Foo, O. H. Franco, A. Franke, B. I. Freedman, S. Freitag-Wolf, Y. Friedlander, P. Froguel, R. T. Gansevoort, H. Gao, P. Gasparini, J. M. Gaziano, V. Giedraitis, C. Gieger, G. Girotto, F. Giulianini, M. Gögele, S. D. Gordon, D. F. Gudbjartsson, V. Gudnason, T. Haller, P. Hamet, T. B. Harris, C. A. Hartman, C. Hayward, J. N. Hellwege, C.-K. Heng, A. A. Hicks, E. Hofer, W. Huang, N. Hutri-Kähönen, S.-J. Hwang, M. A. Ikram, O. S. Indridason, E. Ingelsson, M. Ising, V. W. V. Jaddoe, J. Jakobsdottir, J. B. Jonas, P. K. Joshi, N. S. Josyula, B. Jung, M. Kähönen, Y. Kamatani, C. M. Kammerer, M. Kanai, M. Kastarinen, S. M. Kerr, C.-C. Khor, W. Kiess, M. E. Kleber, W. Koenig, J. S. Kooner, A. Körner, P. Kovacs, A. T. Kraja, A. Krajcoviechova, H. Kramer, B. K. Krämer, F. Kronenberg, M. Kubo, B. Kühnel, M. Kuokkanen, J. Kuusisto, M. L. Bianca, M. Laakso, L. A. Lange, C. D. Langefeld, J. J.-M. Lee, B. Lehne, T. Lehtimäki, W. Lieb, L. C. Study, S.-C. Lim, L. Lind, C. M. Lindgren, J. Liu, J. Liu, M. Loeffler, R. J. F. Loos, S. Lucae, M. A. Lukas, L.-P. Lyytikäinen, R. Mägi, P. K. E. Magnusson, A. Mahajan, N. G. Martin, J. Martins, W. März, D. Mascalzoni, K. Matsuda, C. Meisinger, T. Meitinger, O. Melander, A. Metspalu, E. K. Mikaelsdottir, Y. Milaneschi, K. Miliku, P. P. Mishra; V. A. Million Veteran Program, K. L. Mohlke, N. Mononen, G. W. Montgomery, D. O. Mook-Kanamori, J. C. Mychaleckyj, G. N. Nadkarni, M. A. Nalls, M. Nauck, K. Nikus, B. Ning, I. M. Nolte, R. Noordam, J. O'Connell, M. L. O'Donoghue, I. Olafsson, A. J. Oldehinkel, M. Orho-Melander, W. H. Ouwehand, S. Padmanabhan, N. D. Palmer, R. Palsson, B. W. J. H. Penninx, T. Perls, M. Perola, M. Pirastu, N. Pirastu, G. Pistis, A. I. Podgornaia, O. Polasek, B. Ponte, D. J. Porteous, T. Poulain, P. P. Pramstaller, M. H. Preuss, B. P. Prins, M. A. Province, T. J. Rabelink, L. M. Raffield, O. T. Raitakari, D. F. Reilly, R. Rettig, M. Rheinberger, K. M. Rice, P. M. Ridker, F. Rivadeneira, F. Rizzi, D. J. Roberts, A. Robino, P. Rossing, I. Rudan, R. Rueedi, D. Ruggiero, K. A. Ryan, Y. Saba, C. Sabanayagam, V. Salomaa, E. Salvi, K.-U. Saum, H. Schmidt, R. Schmidt, B. Schöttker, C.-A. Schulz, N. Schupf, C. M. Shaffer, Y. Shi, A. V. Smith, B. H. Smith, N. Soranzo, C. N. Spracklen, K. Strauch, H. M. Stringham, M. Stumvoll, P. O. Svensson, S. Szymczak, E.-S. Tai, S. M. Tajuddin, N. Y. Q. Tan, K. D. Taylor, A.

Teren, Y.-C. Tham, J. Thiery, C. H. L. Thio, H. Thomsen, G. Thorleifsson, D. Toniolo, A. Tönjes, J. Tremblay, I. Tzoulaki, A. G. Uitterlinden, S. Vaccargiu, R. M. van Dam, P. van der Harst, C. M. van Duijn, D. R. Velez Edward, N. Verweij, S. Vogelezang, U. Völker, P. Vollenweider, G. Waeber, M. Waldenberger, L. Wallentin, Y. X. Wang, C. Wang, D. M. Waterworth, W. B. Wei, H. White, J. B. Whitfield, S. H. Wild, J. F. Wilson, M. K. Wojczynski, C. Wong, T.-Y. Wong, L. Xu, Q. Yang, M. Yasuda, L. M. Yerges-Armstrong, W. Zhang, A. B. Zonderman, J. I. Rotter, M. Bochud, B. M. Psaty, V. Vitart, J. G. Wilson, A. Dehghan, A. Parsa, D. I. Chasman, K. Ho, A. P. Morris, O. Devuyst, S. Akilesh, S. A. Pendergrass, X. Sim, C. A. Böger, Y. Okada, T. L. Edwards, H. Snieder, K. Stefansson, A. M. Hung, I. M. Heid, M. Scholz, A. Teumer, A. Köttgen, C. Pattaro, A catalog of genetic loci associated with kidney function from analyses of a million individuals. *Nat. Genet.* **51**, 957–972 (2019).

39. C. A. de Leeuw, J. M. Mooij, T. Heskes, D. Posthuma, MAGMA: Generalized gene-set analysis of GWAS data. *PLOS Comput. Biol.* **11**, e1004219 (2015).
40. I. Everaert, H. De Naeyer, Y. Taes, W. Derave, Gene expression of carnosine-related enzymes and transporters in skeletal muscle. *Eur. J. Appl. Physiol.* **113**, 1169–1179 (2013).
41. M. M. Matthews, T. W. Traut. Regulation of *N*-carbamoyl-β-alanine amidohydrolase, the terminal enzyme in pyrimidine catabolism, by ligand-induced change in polymerization. *J. Biol. Chem.* **262**, 7232–7237 (1987).
42. A. Peracchi The limits of enzyme specificity and the evolution of metabolism. *Trends Biochem. Sci.* **43**, 984–996 (2018).
43. A. Shetewy, K. Shimada-Takaura, D. Warner, C. J. Jong, A.-B. Al Mehdi, M. Alexeyev, K. Takahashi, S. W. Schaffer, Mitochondrial defects associated with β-alanine toxicity: Relevance to hyper-beta-alaninemia. *Mol. Cell. Biochem.* **416**, 11–22 (2016).
44. T. Gemelli, R. B. de Andrade, D. B. Rojas, N. F. Bonorino, P. N. Mazzola, L. S. Tortorelli, C. Funchal, C. S. D. Filho, C. M. D. Wannmacher, Effects of β-alanine administration on selected parameters of oxidative stress and phosphoryltransfer network in cerebral cortex and cerebellum of rats. *Mol. Cell. Biochem.* **380**, 161–170 (2013).

5. M. A. Kamal, H. Jiang, Y. Hu, R. F. Keep, D. E. Smith, Influence of genetic knockout of *Pept2* on the in vivo disposition of endogenous and exogenous carnosine in wild-type and *Pept2* null mice. *Am. J. Physiol. Regul. Integr. Comp. Physiol.* **296**, R986–R991 (2009).

6. E. Miyamoto-Mikami, K. Tsuji, N. Horii, N. Hasegawa, S. Fujie, T. Homma, M. Uchida, T. Hamaoka, H. Kanehisa, I. Tabata, M. Iemitsu, Gene expression profile of muscle adaptation to high-intensity intermittent exercise training in young men. *Sci. Rep.* **8**, 16811 (2018).

47. J. Drozak, M. Veiga-da-Cunha, D. Vertommen, V. Stroobant, E. Van Schaftingen, Molecular identification of carnosine synthase as ATP-grasp domain-containing protein 1 (ATPGD1). *J. Biol. Chem.* **285**, 9346–9356 (2010).

48. B. Janssen, D. Hohenadel, P. Brinkkoetter, V. Peters, N. Rind, C. Fischer, I. Rychlik, M. Cerna, M. Romzova, E. de Heer, H. Baelde, S. J. L. Bakker, M. Zirie, E. Rondeau, P. Mathieson, M. A. Saleem, J. Meyer, H. Köppel, S. Sauerhoefer, C. R. Bartram, P. Nawroth, H.-P. Hammes, B. A. Yard, J. Zschocke, F. J. van der Woude, Carnosine as a protective factor in diabetic nephropathy: Association with a leucine repeat of the carnosinase gene CNDP1. *Diabetes* **54**, 2320–2327 (2005).

49. M. A. Babizhayev, Biochemical, biomedical and metabolic aspects of imidazole-containing dipeptides with the inherent complexity to neurodegenerative diseases and various states of mental well-being: A challenging correction and neurotherapeutic pharmaceutical biotechnology for treating cognitive deficits, depression and intellectual disabilities. *Curr. Pharm. Biotechnol.* **15**, 738–778 (2014).

50. J. Ivanisevic, A. A. Epstein, M. E. Kurczy, P. H. Benton, W. Uritboonthai, H. S. Fox, M. D. Boska, H. E. Gendelman, G. Siuzdak, Brain region mapping using global metabolomics. *Chem. Biol.* **21**, 1575–1584 (2014).

51. M.-C. Senut, S. Azher, F. L. Margolis, K. Patel, A. Mousa, A. Majid, Distribution of carnosine-like peptides in the nervous system of developing and adult zebrafish (*Danio rerio*) and embryonic effects of chronic carnosine exposure. *Cell Tissue Res.* **337**, 45–61 (2009).

2. N. Alenina, D. Kikic, M. Todiras, V. Mosienko, F. Qadri, R. Plehm, P. Boyé, L. Vilianovitch, R. Sohr, K. Tenner, H. Hörtnagl, M. Bader, Growth retardation and altered autonomic control in mice lacking brain serotonin. *Proc. Natl. Acad. Sci. U.S.A.* **106**, 10332–10337 (2009).

53. S. Sun, Y. Sun, S.-C. Ling, L. Ferraiuolo, M. McAlonis-Downes, Y. Zou, K. Drenner, Y. Wang, D. Ditsworth, S. Tokunaga, A. Kopelevich, B. K. Kaspar, C. Lagier-Tourenne, D. W. Cleveland, Translational profiling identifies a cascade of damage initiated in motor neurons

and spreading to glia in mutant SOD1-mediated ALS. *Proc. Natl. Acad. Sci. U.S.A.* **112**, E6993–E7002 (2015).

54. S. Schwartz, E. Hall, G. Ast, SROOGLE: Webserver for integrative, user-friendly visualization of splicing signals. *Nucleic Acids Res.* **37**, W189–W192 (2009).
55. K. J. Livak, T. D. Schmittgen, Analysis of relative gene expression data using real-time quantitative PCR and the $2^{-\Delta\Delta C_T}$ method. *Methods* **25**, 402–408 (2001).
56. D. Kim, B. Langmead, S. L. Salzberg, HISAT: A fast spliced aligner with low memory requirements. *Nat. Methods* **12**, 357–360 (2015).
57. Y. Liao, G. K. Smyth, W. Shi, featureCounts: An efficient general purpose program for assigning sequence reads to genomic features. *Bioinformatics* **30**, 923–930 (2014).
58. M. I. Love, W. Huber, S. Anders, Moderated estimation of fold change and dispersion for RNA-seq data with DESeq2. *Genome Biol.* **15**, 550 (2014).
59. T. Metsalu, J. Vilo, ClustVis: A web tool for visualizing clustering of multivariate data using principal component analysis and heatmap. *Nucleic Acids Res.* **43** (W1), W566–W570 (2015).

0. G. Fenalti, C. S. Hampe, K. O'Connor, J. P. Banga, I. R. Mackay, M. J. Rowley, O. El-Kabbani, Molecular characterization of a disease associated conformational epitope on GAD65 recognised by a human monoclonal antibody b96.11. *Mol. Immunol.* **44**, 1178–1189 (2007).

61. P. Emsley, B. Lohkamp, W G Scott, K. Cowtan, Features and development of Coot. *Acta Crystallogr. D Biol. Crystallogr.* **66** (Pt 4), 486–501 (2010).
62. E. F. Pettersen, T. D. Goddard, C. C. Huang, G. S. Couch, D. M. Greenblatt, E. C. Meng, T. E. Ferrin, UCSF Chimera—A visualization system for exploratory research and analysis. *J. Comput. Chem.* **25**, 1605–1612 (2004).
63. C. G. Langendorf, K. L. Tuck, T. L. G. Key, G. Fenalti, R. N. Pike, C. J. Rosado, A. S. M. Wong, A. M. Buckle, R. H. P. Law, J. C. Whisstock, Structural characterization of the mechanism through which human glutamic acid decarboxylase auto-activates. *Biosci. Rep.* **33**, 137–144 (2013).
64. D. Demontis, R. K. Walters, J. Martin, M. Mattheisen, T. D. Als, E. Agerbo, G. Baldursson, R. Belliveau, J. Bybjerg-Grauholm, M. Bækvad-Hansen, F. Cerrato, K. Chambert, C. Churchhouse, A. Dumont, N. Eriksson, M. Gandal, J. I. Goldstein, K. L. Grasby, J. Grove, O.

O. Gudmundsson, C. S. Hansen, M. E. Hauberg, M. V. Hollegaard, D. P. Howrigan, H. Huang, J. B. Maller, A. R. Martin, N. G. Martin, J. Moran, J. Pallesen, D. S. Palmer, C. B. Pedersen, M. G. Pedersen, T. Poterba, J. B. Poulsen, S. Ripke, E. B. Robinson, F. K. Satterstrom, H. Stefansson, C. Stevens, P. Turley, G. B. Walters, H. Won, M. J. Wright; ADHD Working Group of the Psychiatric Genomics Consortium (PGC); Early Lifecourse & Genetic Epidemiology (EAGLE) Consortium; 23andMe Research Team, O. A. Andreassen, P. Asherson, C. L. Burton, D. I. Boomsma, B. Cormand, S. Dalsgaard, B. Franke, J. Gelernter, D. Geschwind, H. Hakonarson, J. Haavik, H. R. Kranzler, J. Kuntsi, K. Langley, K.-P. Lesch, C. Middeldorp, A. Reif, L. A. Rohde, P. Roussos, R. Schachar, P. Sklar, E. J. S. Sonuga-Barke, P. F. Sullivan, A. Thapar, J. Y. Tung, I. D. Waldman, S. E. Medland, K. Stefansson, M. Nordentoft, D. M. Hougaard, T. Werge, O. Mors, P. B. Mortensen, M. J. Daly, S. V. Faraone, A. D. Børglum, B. M. Neale, Discovery of the first genome-wide significant risk loci for attention deficit/hyperactivity disorder. *Nat. Genet.* **51**, 63–75 (2019).

65. L. G. Fritsche, W. Igl, J. N. C. Bailey, F. Grassmann, S. Sengupta, J. L. Bragg-Gresham, K. P. Burdon, S. J. Hebbring, C. Wen, M. Gorski, I. K. Kim, D. Cho, D. Zack, E. Souied, H. P. N. Scholl, E. Bala, K. E. Lee, D. J. Hunter, R. J. Sardell, P. Mitchell, J. E. Merriam, V. Cipriani, J. D. Hoffman, T. Schick, Y. T. E. Lechanteur, R. H. Guymer, M. P. Johnson, Y. Jiang, C. M. Stanton, G. H. S. Buitendijk, X. Zhan, A. M. Kwong, A. Boleda, M. Brooks, L. Gieser, R. Ratnapriya, K. E. Branham, J. R. Foerster, J. R. Heckenlively, M. I. Othman, B. J. Vote, H. H. Liang, E. Souzeau, I. L. McAllister, T. Isaacs, J. Hall, S. Lake, D. A. Mackey, I. J. Constable, J. E. Craig, T. E. Kitchner, Z. Yang, Z. Su, H. Luo, D. Chen, H. Ouyang, K. Flagg, D. Lin, G. Mao, H. Ferreyra, K. Stark, C. N von Strachwitz, A. Wolf, C. Brandl, G. Rudolph, M. Olden, M. A. Morrison, D. J. Morgan, M. Schu, J. Ahn, G. Silvestri, E. E. Tsironi, K. H. Park, L. A. Farrer, A. Orlin, A. Brucker, M. Li, C. A. Curcio, S. Mohand-Saïd, J.-A. Sahel, I. Audo, M. Benchaboune, A. J. Cree, C. A. Rennie, S. V. Goverdhan, M. Grunin, S. Hagbi-Levi, P. Campochiaro, N. Katsanis, F. G. Holz, F. Blond, H. Blanché, J.-F. Deleuze, R. P. Igo Jr., B. Truitt, N. S. Peachey, S. M. Meuer, C. E. Myers, E. L. Moore, R. Klein, M. A. Hauser, E. A. Postel, M. D. Courtenay, S. G. Schwartz, J. L. Kovach, W. K. Scott, G. Liew, A. G. Tan, B. Gopinath, J. C. Merriam, R. T. Smith, J. C. Khan, H. Shahid, A. T. Moore, J. A. McGrath, R. Laux, M. A. Brantley Jr., A. Agarwal, L. Ersoy, A. Caramoy, T. Langmann, N. T. M. Saksens, E. K. de Jong, C. B. Hoyng, M. S. Cain, A. J. Richardson,

T. M. Martin, J. Blangero, D. E. Weeks, B. Dhillon, C. M van Duijn, K. F. Doheny, J. Romm, C. C. W. Klaver, C. Hayward, M. B. Gorin, M. L. Klein, P. N. Baird, A. I den Hollander, S. Fauser, J. R. W. Yates, R. Allikmets, J. J. Wang, D. A. Schaumberg, B. E. K. Klein, S. A. Hagstrom, I. Chowers, A. J. Lotery, T. Léveillard, K. Zhang, M. H. Brilliant, A. W. Hewitt, A. Swaroop, E. Y. Chew, M. A. Pericak-Vance, M. De Angelis, D. Stambolian, J. L. Haines, S. K. Iyengar, B. H. F. Weber, G. R. Abecasis, I. M. Heid, A large genome-wide association study of age-related macular degeneration highlights contributions of rare and common variants. *Nat. Genet.* **48**, 134–143 (2016).

66. J. Grove, S. Ripke, T. D. Als, M. Mattheisen, R. K. Walters, H. Won, J. Pallesen, E. Agerbo, O. A. Andreassen, R. Anney, S. Awashti, R. Belliveau, F. Bettella, J. D. Buxbaum, J. Bybjerg-Grauholm, M. Bækvad-Hansen, F. Cerrato, K. Chambert, J. H. Christensen, C. Churchhouse, K. Dellenvall, D. Demontis, S. De Rubeis, B. Devlin, S. Djurovic, A. L. Dumont, J. I. Goldstein, C. S. Hansen, M. E. Hauberg, M. V. Hollegaard, S. Hope, D. P. Howrigan, H. Huang, C. M. Hultman, L. Klei, J. Maller, J. Martin, A. R. Martin, J. L. Moran, M. Nyegaard, T. Nærland, D. S. Palmer, A. Palotie, C. B. Pedersen, M. G. Pedersen, T. dPoterba, J. B. Poulsen, B. S. Pourcain, P. Qvist, K. Rehnström, A. Reichenberg, J. Reichert, E. B. Robinson, K. Roeder, P. Roussos, E. Saemundsen, S. Sandin, F. K. Satterstrom, G. D. Smith, H. Stefansson, S. Steinberg, C. R. Stevens, P. F. Sullivan, P. Turley, G. B. Walters, X. Xu; Autism Spectrum Disorder Working Group of the Psychiatric Genomics Consortium; BUPGEN; Major Depressive Disorder Working Group of the Psychiatric Genomics Consortium; 23andMe Research Team, K. Stefansson, D. H. Geschwind, M. Nordentoft, D. M. Hougaard, T. Werge, O. Mors, P. B. Mortensen, B. M. Neale, M. J. Daly, A. D. Børglum, Identification of common genetic risk variants for autism spectrum disorder. *Nat. Genet.* **51**, 431–444 (2019).

67. I. E. Jansen, J. E. Savage, K. Watanabe, J. Bryois, D. M. Williams, S. Steinberg, J. Sealock, I. K. Karlsson, S. Hägg, L. Athanasiu, N. Voyle, P. Proitsi, A. Witoelar, S. Stringer, D. Aarsland, I. S. Almdahl, F. Andersen, S. Bergh, F. Bettella, S. Bjornsson, A. Brækhus, G. Bråthen, C. de Leeuw, R. S. Desikan, S. Djurovic, L. Dumitrescu, T. Fladby, T. J. Hohman, P. V. Jonsson, S. J. Kiddle, A. Rongve, I. Saltvedt, S. B. Sando, G. Selbæk, M. Shoai, N. G. Skene, J. Snaedal, E. Stordal, I. D. Ulstein, Y. Wang, L. R. White, J. Hardy, J. Hjerling-Leffler, P. F. Sullivan, W. M. van der Flier, R. Dobson, L. K. Davis, H. Stefansson, K.

Stefansson, N. L. Pedersen, S. Ripke, O. A. Andreassen, D. Posthuma, Genome-wide meta-analysis identifies new loci and functional pathways influencing Alzheimer's disease risk. *Nat. Genet.* **51**, 404–413 (2019).

68. J. J. Lee, R. Wedow, A. Okbay, E. Kong, O. Maghzian, M. Zacher, T. A. Nguyen-Viet, P. Bowers, J. Sidorenko, R. K. Linnér, M. A. Fontana, T. Kundu, C. Lee, H. Li, R. Li, R. Royer, P. N. Timshel, R. K. Walters, E. A. Willoughby, L. Yengo; 23andMe Research Team; COGENT (Cognitive Genomics Consortium); Social Science Genetic Association Consortium, M. Alver, Y. Bao, D. W. Clark, F. R. Day, N. A. Furlotte, P. K. Joshi, K. E. Kemper, A. Kleinman, C. Langenberg, R. Mägi, J. W. Trampush, S. S. Verma, Y. Wu, M. Lam, J. H. Zhao, Z. Zheng, J. D. Boardman, H. Campbell, J. Freese, K. M. Harris, C. Hayward, P. Herd, M. Kumari, T. Lencz, J. Luan, A. K. Malhotra, A. Metspalu, L. Milani, K. K. Ong, J. R. B. Perry, D. J. Porteous, M. D. Ritchie, M. C. Smart, B. H. Smith, J. Y. Tung, N. J. Wareham, J. F. Wilson, J. P. Beauchamp, D. C. Conley, T. Esko, S. F. Lehrer, P. K. E. Magnusson, S. Oskarsson, T. H. Pers, M. R. Robinson, K. Thom, C. Watson, C. F. Chabris, M. N. Meyer, D. I. Laibson, J. Yang, M. Johannesson, P. D. Koellinger, P. Turley, P. M. Visscher, D. J. Benjamin, D. Cesarini, Gene discovery and polygenic prediction from a genome-wide association study of educational attainment in 1.1 million individuals. *Nat. Genet.* **50**, 1112–1121 (2018).
69. M. A. Nalls, C. Blauwendraat, C. L. Vallerga, K. Heilbron, S. Bandres-Ciga, D. Chang, M. Tan, D. A. Kia, A. J. Noyce, A. Xue, J. Bras, E. Young, R. von Coelln, J. Simón-Sánchez, C. Schulte, M. Sharma, L. Krohn, L. Pihlstrøm, A. Siitonen, H. Iwaki, H. Leonard, F. Faghri, J. R. Gibbs, D. G. Hernandez, S. W. Scholz, J. A. Botia, M. Martinez, J.-C. Corvol, S. Lesage, J. Jankovic, L. M. Shulman, M. Sutherland, P. Tienari, K. Majamaa, M. Toft, O. A. Andreassen, T. Bangale, A. Brice, J. Yang, Z. Gan-Or, T. Gasser, P. Heutink, J. M. Shulman, N. W. Wood, D. A. Hinds, J. A. Hardy, H. R. Morris, J. Gratten, P. M. Visscher, R. R. Graham, A. B. Singleton; 23andMe Research Team; System Genomics of Parkinson’s Disease Consortium; International Parkinson’s Disease Genomics Consortium, Identification of novel risk loci, causal insights, and heritable risk for Parkinson's disease: A meta-analysis of genome-wide association studies. *Lancet Neurol.* **18**, 1091–1102 (2019).
70. A. Nicolas, K. P. Kenna, A. E. Renton, N. Ticozzi, F. Faghri, R. Chia, J. A. Dominov, B. J. Kenna, M. A. Nalls, P. Keagle, A. M. Rivera, W. van Rheenen, N. A. Murphy, J. J. F. A. van

Vugt, J. T. Geiger, R. A. Van der Spek, H. A. Pliner, Shankaracharya, B. N. Smith, G. Marangi, S. D. Topp, Y. Abramzon, A. S. Gkazi, J. D. Eicher, A. Kenna; ITALSGEN Consortium, G. Mora, A. Calvo, L. Mazzini, N. Riva, J. Mandrioli, C. Caponnetto, S. Battistini, P. Volanti, V. L. Bella, F. L. Conforti, G. Borghero, S. Messina, I. L. Simone, F. Trojsi, F. Salvi, F. O. Logullo, S. D'Alfonso, L. Corrado, M. Capasso, L. Ferrucci; Genomic Translation for ALS Care (GTAC) Consortium, C. de Araujo Martins Moreno, S. Kamalakaran, D. B. Goldstein; ALS Sequencing Consortium, A. D. Gitler, T. Harris, R. M. Myers; NYGC ALS Consortium, H. Phatnani, R. L. Musunuri, U. S. Evani, A. Abhyankar, M. C. Zody; Answer ALS Foundation, J. Kaye, S. Finkbeiner, S. K. Wyman, A. L. Nail, L. Lima, E. Fraenkel, C. N. Svendsen, L. M. Thompson, J. E. Van Eyk, J. D. Berry, T. M. Miller, S. J. Kolb, M. Cudkowicz, E. Baxi; Clinical Research in ALS and Related Disorders for Therapeutic Development (CRe ATe) Consortium, M. Benatar, J Paul Taylor, E. Rampersaud, G. Wu, J. Wuu; SLAGEN Consortium, G. Lauria, F. Verde, I. Fogh, C. Tiloca, G. P. Comi, G. Sorarù, C. Cereda; French ALS Consortium, P. Corcia, H. Laaksovirta, L. Myllykangas, L. Jansson, M. Valori, J. Ealing, H. Hamdalla, S. Rollinson, S. Pickering-Brown, R. W. Orrell, K. C. Sidle, A. Malaspina, J. Hardy, A. B. Singleton, J. O. Johnson, S. Arepalli, P. C. Sapp, D. McKenna-Yasek, M. Polak, S. Asress, S. Al-Sarraj, A. King, C. Troakes, C. Vance, J. de Belleroche, F. Baas, A. L. M. A. Ten Asbroek, J. L. Muñoz-Blanco, D. G. Hernandez, J. Ding, J. R. Gibbs, S. W. Scholz, M. K. Floeter, R. H. Campbell, F. Landi, R. Bowser, S. M. Pulst, J. M. Ravits, Daniel J L Mac Gowan, J. Kirby, E. P. Pioro, R. Pamphlett, J. Broach, G. Gerhard, T. L. Dunckley, C. B. Brady, N. W. Kowall, J. C. Troncoso, I. L. Ber, K. Mouzat, S. Lumbroso, T. D. Heiman-Patterson, F. Kamel, L. Van Den Bosch, R. H. Baloh, T. M. Strom, T. Meitinger, A. Shatunov, K. R. Van Eijk, M. de Carvalho, M. Kooyman, B. Middelkoop, M. Moisse, R. L. Mc Laughlin, M. A. Van Es, M. Weber, K. B. Boylan, M. Van Blitterswijk, R. Rademakers, K. E. Morrison, A. N. Basak, J. S. Mora, V. E. Drory, P. J. Shaw, M. R. Turner, K. Talbot, O. Hardiman, K. L. Williams, J. A. Fifita, G. A. Nicholson, I. P. Blair, G. A. Rouleau, J. Esteban-Pérez, A. García-Redondo, A. Al-Chalabi; Project MinE ALS Sequencing Consortium, E. Rogaeva, L. Zinman, L. W. Ostrow, N. J. Maragakis, J. D. Rothstein, Z. Simmons, J. Cooper-Knock, A. Brice, S. A. Goutman, E. L. Feldman, S. B. Gibson, F. Taroni, A. Ratti, C. Gellera, P. Van Damme, W. Robberecht, P. Fratta, M. Sabatelli, C. Lunetta, A. C. Ludolph, P. M. Andersen, J. H.

Weishaupt, W. Camu, J. Q. Trojanowski, V. M. Van Deerlin, R. H. Brown Jr., L. H. van den Berg, J. H. Veldink, M. B. Harms, J. D. Glass, D. J. Stone, P. Tienari, V. Silani, A. Chiò, C. E. Shaw, B. J. Traynor, J. E. Landers, Genome-wide analyses identify KIF5A as a novel ALS gene. *Neuron* **97**, 1268–1283.e6 (2018).

71. T. Otowa, K. Hek, M. Lee, E. M. Byrne, S. S. Mirza, M. G. Nivard, T. Bigdeli, S. H. Aggen, D. Adkins, A. Wolen, A. Fanous, M. C. Keller, E. Castelao, Z. Kutalik, S. Van der Auwera, G. Homuth, M. Nauck, A. Teumer, Y. Milaneschi, J.-J. Hottenga, N. Direk, A. Hofman, A. Uitterlinden, C. L. Mulder, A. K. Henders, S. E. Medland, S. Gordon, A. C. Heath, P. A. F. Madden, M. L. Pergadia, P. J. van der Most, I. M. Nolte, F. V. A. van Oort, C. A. Hartman, A. J. Oldehinkel, M. Preisig, H. J. Grabe, C. M. Middeldorp, B. W. J. H. Penninx, D. Boomsma, N. G. Martin, G. Montgomery, B. S. Maher, E. J. van den Oord, N. R. Wray, H. Tiemeier, J. M. Hettema, Meta-analysis of genome-wide association studies of anxiety disorders. *Mol. Psychiatry* **21**, 1485 (2016).

72. E. A. Stahl, G. Breen, A. J. Forstner, A. M. Quillin, S. Ripke, V. Trubetskoy, M. Mattheisen, Y. Wang, J. R. I. Coleman, H. A. Gaspar, C. A. de Leeuw, S. Steinberg, J. M. Whitehead Pavlides, M. Trzaskowski, E. M. Byrne, T. H. Pers, P. A. Holmans, A. L. Richards, L. Abbott, E. Agerbo, H. Akil, D. Albani, N. Alliey-Rodriguez, T. D. Als, A. Anjorin, V. Antilla, S. Awasthi, J. A. Badner, M. Bækvad-Hansen, J. D. Barchas, N. Bass, M. Bauer, R. Belliveau, S. E. Bergen, C. B. Pedersen, E. Bøen, M. P. Boks, J. Boocock, M. Budde, W. Bunney, M. Burmeister, J. Bybjerg-Grauholm, W. Byerley, M. Casas, F. Cerrato, P. Cervantes, K. Chambert, A. W. Charney, D. Chen, C. Churchhouse, T.-K. Clarke, W. Coryell, D. W. Craig, C. Cruceanu, D. Curtis, P. M. Czerski, A. M. Dale, S. de Jong, F. Degenhardt, J. Del-Favero, J. R. De Paulo, S. Djurovic, A. L. Dobbyn, A. Dumont, T. Elvsåshagen, V. Escott-Price, C. C. Fan, S. B. Fischer, M. Flickinger, T. M. Foroud, L. Forty, J. Frank, C. Fraser, N. B. Freimer, L. Frisén, K. Gade, D. Gage, J. Garnham, C. Giambartolomei, M. G. Pedersen, J. Goldstein, S. D. Gordon, K. Gordon-Smith, E. K. Green, M. J. Green, T. A. Greenwood, J. Grove, W. Guan, J. Guzman-Parra, M. L. Hamshere, M. Hautzinger, U. Heilbronner, S. Herms, M. Hipolito, P. Hoffmann, D. Holland, L. Huckins, S. Jamain, J. S. Johnson, A. Juréus, R. Kandaswamy, R. Karlsson, J. L. Kennedy, S. Kittel-Schneider, J. A. Knowles, M. Kogevinas, A. C. Koller, R. Kupka, C. Lavebratt, J. Lawrence, W. B. Lawson, M. Leber, P. H. Lee, S. E. Levy, J. Z. Li, C. Liu, S. Lucae, A. Maaser, D. J.

Mac Intyre, P. B. Mahon, W. Maier, L. Martinsson, S. M. Carroll, P. M. Guffin, M. G. McInnis, J. D. Mc Kay, H. Medeiros, S. E. Medland, F. Meng, L. Milani, G. W. Montgomery, D. W. Morris, T. W. Mühleisen, N. Mullins, H. Nguyen, C. M. Nievergelt, A. N. Adolfsson, E. A. Nwulia, C. O'Donovan, L. M. Olde Loohuis, A. P. S. Ori, L. Oruc, U. Ösby, R. H. Perlis, A. Perry, A. Pfennig, J. B. Potash, S. M. Purcell, E. J. Regeer, A. Reif, C. S. Reinbold, J. P. Rice, F. Rivas, M. Rivera, P. Roussos, D. M. Ruderfer, E. Ryu, C. Sánchez-Mora, A. F. Schatzberg, W. A. Scheftner, N. J. Schork, C. S. Weickert, T. Shehktman, P. D. Shilling, E. Sigurdsson, C. Slaney, O. B. Smeland, J. L. Sobell, C. S. Hansen, A. T. Spijker, D. S. Clair, M. Steffens, J. S. Strauss, F. Streit, J. Strohmaier, S. Szelinger, R. C. Thompson, T. E. Thorgeirsson, J. Treutlein, H. Vedder, W. Wang, S. J. Watson, T. W. Weickert, S. H. Witt, S. Xi, W. Xu, A. H. Young, P. Zandi, P. Zhang, S. Zöllner; eQTLGen Consortium; BIOS Consortium, R. Adolfsson, I. Agartz, M. Alda, L. Backlund, B. T. Baune, F. Bellivier, W. H. Berrettini, J. M. Biernacka, D. H. R. Blackwood, M. Boehnke, A. D. Børglum, A. Corvin, N. Craddock, M. J. Daly, U. Dannlowski, T. Esko, B. Etain, M. Frye, J. M. Fullerton, E. S. Gershon, M. Gill, F. Goes, M. Grigoroiu-Serbanescu, J. Hauser, D. M. Hougaard, C. M. Hultman, I. Jones, L. A. Jones, R. S. Kahn, G. Kirov, M. Landén, M. Leboyer, C. M. Lewis, Q. S. Li, J. Lissowska, N. G. Martin, F. Mayoral, S. L. Mc Elroy, A. M. McIntosh, F. J. McMahon, I. Melle, A. Metspalu, P. B. Mitchell, G. Morken, O. Mors, P. B. Mortensen, B. Müller-Myhsok, R. M. Myers, B. M. Neale, V. Nimgaonkar, M. Nordentoft, M. M. Nöthen, M. C. O'Donovan, K. J. Oedegaard, M. J. Owen, S. A. Paciga, C. Pato, M. T. Pato, D. Posthuma, J. A. Ramos-Quiroga, M. Ribasés, M. Rietschel, G. A. Rouleau, M. Schalling, P. R. Schofield, T. G. Schulze, A. Serretti, J. W. Smoller, H. Stefansson, K. Stefansson, E. Stordal, P. F. Sullivan, G. Turecki, A. E. Vaaler, E. Vieta, J. B. Vincent, T. Werge, J. I. Nurnberger, N. R. Wray, A. D. Florio, H. J. Edenberg, S. Cichon, R. A. Ophoff, L. J. Scott, O. A. Andreassen, J. Kelsoe, P. Sklar; the Bipolar Disorder Working Group of the Psychiatric Genomics Consortium, Genome-wide association study identifies 30 loci associated with bipolar disorder. *Nat. Genet.* **51**, 793–803 (2019).

73. A. Teumer, A. Tin, R. Sorice, M. Gorski, N. C. Yeo, A. Y. Chu, M. Li, Y. Li, V. Mijatovic, Y.-A. Ko, D. Taliun, A. Luciani, M.-H. Chen, Q. Yang, M. C. Foster, M. Olden, L. T. Hiraki, B. O. Tayo, C. Fuchsberger, A. K. Dieffenbach, A. R. Shuldiner, A. V. Smith, A. M. Zappa,

A. Lupo, B. Kollerits, B. Ponte, B. Stengel, B. K. Krämer, B. Paulweber, B. D. Mitchell, C. Hayward, C. Helmer, C. Meisinger, C. Gieger, C. M. Shaffer, C. Müller, C. Langenberg, D. Ackermann, D. Siscovick; DCCT/EDIC, E. Boerwinkle, F. Kronenberg, G. B. Ehret, G. Homuth, G. Waeber, G. Navis, G. Gambaro, G. Malerba, G. Eiriksdottir, G. Li, H. E. Wichmann, H. Grallert, H. Wallaschofski, H. Völzke, H. Brenner, H. Kramer, I. M. Leach, I. Rudan, H. L. Hillege, J. S. Beckmann, J. C. Lambert, J. Luan, J. H. Zhao, J. Chalmers, J. Coresh, J. C. Denny, K. Butterbach, L. J. Launer, L. Ferrucci, L. Kedenko, M. Haun, M. Metzger, M. Woodward, M. J. Hoffman, M. Nauck, M. Waldenberger, M. Pruijm, M. Bochud, M. Rheinberger, N. Verweij, N. J. Wareham, N. Endlich, N. Soranzo, O. Polasek, P. van der Harst, P. P. Pramstaller, P. Vollenweider, P. S. Wild, R. T. Gansevoort, R. Rettig, R. Biffar, R. J. Carroll, R. Katz, R. J. F. Loos, S.-J. Hwang, S. Coassin, S. Bergmann, S. E. Rosas, S. Stracke, T. B. Harris, T. Corre, T. Zeller, T. Illig, T. Aspelund, T. Tanaka, U. Lendeckel, U. Völker, V. Gudnason, V. Chouraki, W. Koenig, Z. Kutalik, J. R O'Connell, A. Parsa, I. M. Heid, A. D. Paterson, I. H. de Boer, O. Devuyst, J. Lazar, K. Endlich, K. Susztak, J. Tremblay, P. Hamet, H. J. Jacob, C. A. Böger, C. S. Fox, C. Pattaro, A. Köttgen, Genome-wide association studies identify genetic loci associated with albuminuria in diabetes. *Diabetes* **65**, 803–817 (2016).

74. P. van der Harst, N. Verweij, Identification of 64 novel genetic loci provides an expanded view on the genetic architecture of coronary artery disease. *Circ. Res.* **122**, 433–443 (2018).
75. H. J. Watson, Z. Yilmaz, L. M. Thornton, C. Hübel, J. R. I. Coleman, H. A. Gaspar, J. Bryois, A. Hinney, V. M. Leppä, M. Mattheisen, S. E. Medland, S. Ripke, S. Yao, P. Giusti-Rodríguez; Anorexia Nervosa Genetics Initiative, K. B. Hanscombe, K. L. Purves; Eating Disorders Working Group of the Psychiatric Genomics Consortium, R. A. H. Adan, L. Alfredsson, T. Ando, O. A. Andreassen, J. H. Baker, W. H. Berrettini, I. Boehm, C. Boni, V. B. Perica, K. Buehren, R. Burghardt, M. Cassina, S. Cichon, M. Clementi, R. D. Cone, P. Courtet, S. Crow, J. J. Crowley, U. N. Danner, O. S. P. Davis, M. de Zwaan, G. Dedoussis, D. Degortes, J. E. De Socio, D. M. Dick, D. Dikeos, C. Dina, M. Dmitrzak-Weglarz, E. Docampo, L. E. Duncan, K. Egberts, S. Ehrlich, G. Escaramís, T. Esko, X. Estivill, A. Farmer, A. Favaro, F. Fernández-Aranda, M. M. Fichter, K. Fischer, M. Föcker, L. Foretova, A. J. Forstner, M. Forzan, C. S. Franklin, S. Gallinger, I. Giegling, J. Giuranna, F. Gonidakis, P. Gorwood, M. G. Mayora, S. Guillaume, Y. Guo, H. Hakonarson, K. Hatzikotoulas, J.

Hauser, J. Hebebrand, S. G. Helder, S. Herms, B. Herpertz-Dahlmann, W. Herzog, L. M. Huckins, J. I. Hudson, H. Imgart, H. Inoko, V. Janout, S. Jiménez-Murcia, A. Julià, G. Kalsi, D. Kaminská, J. Kaprio, L. Karhunen, A. Karwautz, M. J. H. Kas, J. L. Kennedy, A. Keski-Rahkonen, K. Kiezebrink, Y.-R. Kim, L. Klareskog, K. L. Klump, G. P. S. Knudsen, M. C. La Via, S. L. Hellard, R. D. Levitan, D. Li, L. Lilenfeld, B. D. Lin, J. Lissowska, J. Luykx, P. J. Magistretti, M. Maj, K. Mannik, S. Marsal, C. R. Marshall, M. Mattingsdal, S. M. Devitt, P. M. Guffin, A. Metspalu, I. Meulenbelt, N. Micali, K. Mitchell, A. M. Monteleone, P. Monteleone, M. A. Munn-Chernoff, B. Nacmias, M. Navratilova, I. Ntalla, J. K. O'Toole, R. A. Ophoff, L. Padyukov, A. Palotie, J. Pantel, H. Papezova, D. Pinto, R. Rabionet, A. Raevuori, N. Ramoz, T. Reichborn-Kjennerud, V. Ricca, S. Ripatti, F. Ritschel, M. Roberts, A. Rotondo, D. Rujescu, F. Rybakowski, P. Santonastaso, A. Scherag, S. W. Scherer, U. Schmidt, N. J. Schork, A. Schosser, J. Seitz, L. Slachtova, P. E. Slagboom, M. C. T. Slof-Op 't Landt, A. Slopien, S. Sorbi, B. Świątkowska, J. P. Szatkiewicz, I. Tachmazidou, E. Tenconi, A. Tortorella, F. Tozzi, J. Treasure, A. Tsitsika, M. Tyszkiewicz-Nwafor, K. Tziouvas, A. A. van Elburg, E. F. van Furth, G. Wagner, E. Walton, E. Widen, E. Zeggini, S. Zerwas, S. Zipfel, A. W. Bergen, J. M. Boden, H. Brandt, S. Crawford, K. A. Halmi, L. John Horwood, C. Johnson, A. S. Kaplan, W. H. Kaye, J. E. Mitchell, C. M. Olsen, J. F. Pearson, N. L. Pedersen, M. Strober, T. Werge, D. C. Whiteman, D. B. Woodside, G. D. Stuber, S. Gordon, J. Grove, A. K. Henders, A. Juréus, K. M. Kirk, J. T. Larsen, R. Parker, L. Petersen, J. Jordan, M. Kennedy, G. W. Montgomery, T. D. Wade, A. Birgegård, P. Lichtenstein, C. Norring, M. Landén, N. G. Martin, P. B. Mortensen, P. F. Sullivan, G. Breen, C. M. Bulik, Genome-wide association study identifies eight risk loci and implicates metabo-psychiatric origins for anorexia nervosa. *Nat. Genet.* **51**, 1207–1214 (2019).

76. N. R. Wray, S. Ripke, M. Mattheisen, M. Trzaskowski, E. M. Byrne, A. Abdellaoui, M. J. Adams, E. Agerbo, T. M. Air, T. M. F. Andlauer, S.-A. Bacanu, M. Bækvad-Hansen, A. F. T. Beekman, T. B. Bigdeli, E. B. Binder, D. R. H. Blackwood, J. Bryois, H. N. Buttenschøn, J. Bybjerg-Grauholm, N. Cai, E. Castelao, J. H. Christensen, T.-K. Clarke, J. I. R. Coleman, L. Colodro-Conde, B. Couvy-Duchesne, N. Craddock, G. E. Crawford, C. A. Crowley, H. S. Dashti, G. Davies, I. J. Deary, F. Degenhardt, E. M. Derks, N. Direk, C. V. Dolan, E. C. Dunn, T. C. Eley, N. Eriksson, V. Escott-Price, F. H. F. Kiadeh, H. K. Finucane, A. J. Forstner, J. Frank, H. A. Gaspar, M. Gill, P. Giusti-Rodríguez, F. S. Goes, S. D. Gordon, J.

Grove, L. S. Hall, E. Hannon, C. S. Hansen, T. F. Hansen, S. Herms, I. B. Hickie, P. Hoffmann, G. Homuth, C. Horn, J.-J. Hottenga, D. M. Hougaard, M. Hu, C. L. Hyde, M. Ising, R. Jansen, F. Jin, E. Jorgenson, J. A. Knowles, I. S. Kohane, J. Kraft, W. W. Kretzschmar, J. Krogh, Z. Kutalik, J. M. Lane, Y. Li, Y. Li, P. A. Lind, X. Liu, L. Lu, D. J. MacIntyre, D. F. MacKinnon, R. M. Maier, W. Maier, J. Marchini, H. Mbarek, P. M. Grath, P. M. Guffin, S. E. Medland, D. Mehta, C. M. Middeldorp, E. Mihailov, Y. Milaneschi, L. Milani, J. Mill, F. M. Mondimore, G. W. Montgomery, S. Mostafavi, N. Mullins, M. Nauck, B. Ng, M. G. Nivard, D. R. Nyholt, P. F. O'Reilly, H. Oskarsson, M. J. Owen, J. N. Painter, C. B. Pedersen, M. G. Pedersen, R. E. Peterson, E. Pettersson, W. J. Peyrot, G. Pistis, D. Posthuma, S. M. Purcell, J. A. Quiroz, P. Qvist, J. P. Rice, B. P. Riley, M. Rivera, S. S. Mirza, R. Saxena, R. Schoevers, E. C. Schulte, L. Shen, J. Shi, S. I. Shyn, E. Sigurdsson, G. B. C. Sinnamon, J. H. Smit, D. J. Smith, H. Stefansson, S. Steinberg, C. A. Stockmeier, F. Streit, J. Strohmaier, K. E. Tansey, H. Teismann, A. Teumer, W. Thompson, P. A. Thomson, T. E. Thorgeirsson, C. Tian, M. Traylor, J. Treutlein, V. Trubetskoy, A. G. Uitterlinden, D. Umbricht, S. Van der Auwera, A. M. van Hemert, A. Viktorin, P. M. Visscher, Y. Wang, B. T. Webb, S. M. Weinsheimer, J. Wellmann, G. Willemsen, S. H. Witt, Y. Wu, H. S. Xi, J. Yang, F. Zhang; eQTLGen; 23andMe, V. Arolt, B. T. Baune, K. Berger, D. I. Boomsma, S. Cichon, U. Dannlowski, E. C. J. de Geus, J. R. De Paulo, E. Domenici, K. Domschke, T. Esko, H. J. Grabe, S. P. Hamilton, C. Hayward, A. C. Heath, D. A. Hinds, K. S. Kendler, S. Kloiber, G. Lewis, Q. S. Li, S. Lucae, P. F. A. Madden, P. K. Magnusson, N. G. Martin, A. M. Mc Intosh, A. Metspalu, O. Mors, P. B. Mortensen, B. Müller-Myhsok, M. Nordentoft, M. M. Nöthen, M. C. O'Donovan, S. A. Paciga, N. L. Pedersen, B. W. J. H. Penninx, R. H. Perlis, D. J. Porteous, J. B. Potash, M. Preisig, M. Rietschel, C. Schaefer, T. G. Schulze, J. W. Smoller, K. Stefansson, H. Tiemeier, R. Uher, H. Völzke, M. M. Weissman, T. Werge, A. R. Winslow, C. M. Lewis, D. F. Levinson, G. Breen, A. D. Børglum, P. F. Sullivan; the Major Depressive Disorder Working Group of the Psychiatric Genomics Consortium, Genome-wide association analyses identify 44 risk variants and refine the genetic architecture of major depression. *Nat. Genet.* **50**, 668–681 (2018).

77. A. Xue, Y. Wu, Z. Zhu, F. Zhang, K. E. Kemper, Z. Zheng, L. Yengo, L. R. Lloyd-Jones, J. Sidorenko, Y. Wu; eQTLGen Consortium, A. F. Mc Rae, P. M. Visscher, J. Zeng, J. Yang,

Genome-wide association analyses identify 143 risk variants and putative regulatory mechanisms for type 2 diabetes. *Nat. Commun.* **9**, 2941 (2018).

78. L. Yengo, J. Sidorenko, K. E. Kemper, Z. Zheng, A. R. Wood, M. N. Weedon, T. M. Frayling, J. Hirschhorn, J. Yang, P. M. Visscher; GIANT Consortium, Meta-analysis of genome-wide association studies for height and body mass index in approximately 700000 individuals of European ancestry. *Hum. Mol. Genet.* **27**, 3641–3649 (2018).
79. International League Against Epilepsy Consortium on Complex Epilepsies, Genetic determinants of common epilepsies: A meta-analysis of genome-wide association studies. *Lancet Neurol.* **13**, 893–903 (2014).
80. A. Okbay, B. M. L. Baselmans, J.-E. De Neve, P. Turley, M. G. Nivard, M. A. Fontana, S. F. W. Meddens, R. K. Linnér, C. A. Rietveld, J. Derringer, J. Gratten, J. J. Lee, J. Z. Liu, R. de Vlaming, T. S. Ahluwalia, J. Buchwald, A. Cavadino, A. C. Frazier-Wood, N. A. Furlotte, V. Garfield, M. H. Geisel, J. R. Gonzalez, S. Haitjema, R. Karlsson, Sander W van der Laan, K.-H. Ladwig, J. Lahti, S. J. van der Lee, P. A. Lind, T. Liu, L. Matteson, E. Mihailov, M. B. Miller, C. C. Minica, I. M. Nolte, D. Mook-Kanamori, P. J. van der Most, C. Oldmeadow, Y. Qian, O. Raitakari, R. Rawal, A. Realo, R. Rueedi, B. Schmidt, A. V. Smith, E. Stergiakouli, T. Tanaka, K. Taylor, G. Thorleifsson, J. Wedenoja, J. Wellmann, H.-J. Westra, S. M. Willems, W. Zhao; Life Lines Cohort Study, N. Amin, A. Bakshi, S. Bergmann, G. Bjornsdottir, P. A. Boyle, S. Cherney, S. R. Cox, G. Davies, O. S. P. Davis, J. Ding, N. Direk, P. Eibich, R. T. Emeny, G. Fatemifar, J. D. Faul, L. Ferrucci, A. J. Forstner, C. Gieger, R. Gupta, T. B. Harris, J. M. Harris, E. G. Holliday, J.-J. Hottenga, P. L. De Jager, M. A. Kaakinen, E. Kajantie, V. Karhunen, I. Kolcic, M. Kumari, L. J. Launer, L. Franke, R. Li-Gao, D. C. Liewald, M. Koini, A. Loukola, P. Marques-Vidal, G. W. Montgomery, M. A. Mosing, L. Paternoster, A. Pattie, K. E. Petrovic, L. Pulkki-Råback, L. Quaye, K. Räikkönen, I. Rudan, R. J. Scott, J. A. Smith, A. R. Sutin, M. Trzaskowski, A. E. Vinkhuyzen, L. Yu, D. Zabaneh, J. R. Attia, D. A. Bennett, K. Berger, L. Bertram, D. I. Boomsma, H. Snieder, S.-C. Chang, F. Cucca, I. J. Deary, C. M. van Duijn, J. G. Eriksson, U. Bültmann, E. J. C. de Geus, P. J. F. Groenen, V. Gudnason, T. Hansen, C. A. Hartman, C. M. A. Haworth, C. Hayward, A. C. Heath, D. A. Hinds, E. Hyppönen, W. G. Iacono, M.-R. Järvelin, K.-H. Jöckel, J. Kaprio, S. L. R. Kardia, L. Keltikangas-Järvinen, P. Kraft, L. D. Kubzansky, T. Lehtimäki, P. K. E. Magnusson, N. G. Martin, M. M. Gue, A. Metspalu, M. Mills, R. de Mutsert, A. J.

Oldehinkel, G. Pasterkamp, N. L. Pedersen, R. Plomin, O. Polasek, C. Power, S. S. Rich, F. R. Rosendaal, H. M. den Ruijter, D. Schlessinger, H. Schmidt, R. Svento, R. Schmidt, B. Z. Alizadeh, T. I. A. Sørensen, T. D. Spector, J. M. Starr, K. Stefansson, A. Steptoe, A. Terracciano, U. Thorsteinsdottir, A. R. Thurik, N. J. Timpson, H. Tiemeier, A. G. Uitterlinden, P. Vollenweider, G. G. Wagner, D. R. Weir, J. Yang, D. C. Conley, G. D. Smith, A. Hofman, M. Johannesson, D. I. Laibson, S. E. Medland, M. N. Meyer, J. K. Pickrell, T. Esko, R. F. Krueger, J. P. Beauchamp, P. D. Koellinger, D. J. Benjamin, M. Bartels, D. Cesarini, Genetic variants associated with subjective well-being, depressive symptoms, and neuroticism identified through genome-wide analyses. *Nat. Genet.* **48**, 624–633 (2016).

81. Schizophrenia Working Group of the Psychiatric Genomics Consortium, Biological insights from 108 schizophrenia-associated genetic loci. *Nature* **511**, 421–427 (2014).
